# High sink strength prevents photosynthetic down-regulation in cassava grown at elevated CO_2_ concentration

**DOI:** 10.1093/jxb/eraa459

**Published:** 2020-10-12

**Authors:** Ursula M Ruiz-Vera, Amanda P De Souza, Michael R Ament, Roslyn M Gleadow, Donald R Ort

**Affiliations:** 1 Carl R. Woese Institute for Genomic Biology, University of Illinois at Urbana-Champaign, Urbana, IL, USA; 2 School of Biological Sciences, Monash University, Clayton, Victoria, Australia; 3 Departments of Plant Biology and Crop Sciences, University of Illinois at Urbana-Champaign, Urbana, IL, USA; 4 Brookhaven National Laboratory, USA

**Keywords:** African crops, climate change effects on plants, crop improvement, cyanide, food security, photosynthesis, photosynthetic efficiency, root sink capacity, source–sink relationship, staple root crop

## Abstract

Cassava has the potential to alleviate food insecurity in many tropical regions, yet few breeding efforts to increase yield have been made. Improved photosynthetic efficiency in cassava has the potential to increase yields, but cassava roots must have sufficient sink strength to prevent carbohydrates from accumulating in leaf tissue and suppressing photosynthesis. Here, we grew eight farmer-preferred African cassava cultivars under free-air CO_2_ enrichment (FACE) to evaluate the sink strength of cassava roots when photosynthesis increases due to elevated CO_2_ concentrations ([CO_2_]). Relative to the ambient treatments, elevated [CO_2_] treatments increased fresh (+27%) and dry (+37%) root biomass, which was driven by an increase in photosynthesis (+31%) and the absence of photosynthetic down-regulation over the growing season. Moreover, intrinsic water use efficiency improved under elevated [CO_2_] conditions, while leaf protein content and leaf and root cyanide concentrations were not affected. Overall, these results suggest that higher cassava yields can be expected as atmospheric [CO_2_] increases over the coming decades. However, there were cultivar differences in the partitioning of resources to roots versus above-grown biomass; thus, the particular responses of each cultivar must be considered when selecting candidates for improvement.

## Teaser

The high sink strength of cassava storage roots prevented the down-regulation of photosynthesis under elevated CO_2_ concentrations, leading to higher yields.

## Introduction

Cassava (*Manihot esculenta* Crantz.) is a staple food source for >1 billion people (Chetty *et al*., 2013), providing over a quarter of per capita calorie consumption in many food-insecure regions, including sub-Saharan Africa (Nweke, 2005). Cassava’s importance as a food security crop relates to its ability to produce satisfactory yields in marginal environments, to repel herbivores by producing cyanogenic compounds, and to remain edible when harvest is delayed for up to 3 years (El-Sharkawy, 1993; Siritunga and Sayre, 2004; Lebot, 2009). Although tuberous roots are the more popular edible portion of cassava, ~60% of countries in sub-Saharan Africa also consume the leaves. Cassava leaves have a higher protein content than roots and serve as an important protein supplement in the human diet after their detoxification (e.g. pounding with grinding and cooking; Latif and Müller, 2015). Cassava roots and foliage (leaves and stems) are also used as biofuel in Asia and as animal feed in Africa, Asia, and South America (Howeler, 2006; Oppong-Apane, 2013; Marx, 2019).

The demand for cassava is expected to increase over the next decades due to increased population pressure and climate change. For example, in Nigeria, the largest cassava producer in the world, an estimated shortfall of 12 Mt is predicted by 2030 (IITA, 2017). Concerns over future cassava yield shortages are magnified by the fact that cassava storage root yield has not increased significantly in most regions since the 1990s (Ceballos *et al*., 2016; De Souza *et al*., 2017). Although breeding programs have been established to increase cassava yields through improving pest resistance, disease and drought tolerance, and agricultural practices (El-Sharkawy, 2004; Center for Tropical Agriculture, 2007), efforts to directly breed for increased root biomass have slowed in recent years, in part because other breeding objectives (e.g. for high nutrient content or disease resistance) are controlled by fewer genes and are thus perceived as more attainable (Ceballos *et al*., 2016).

Enhancing photosynthetic efficiency has been proposed as a strategy to increase the yield of crops such as cassava (De Souza *et al*., 2017). In the model crop tobacco, improved photosynthetic efficiency has led to 15–25% increases in biomass (Kromdijk *et al*., 2016; South *et al*., 2019). Whether the same success can be achieved in food crops, however, depends on the ability of the crops to utilize the greater carbohydrate pool produced by enhanced photosynthetic rates (Sonnewald and Fernie, 2018), which in turn depends on the coordination between source and sink tissues for carbohydrate production and utilization. Down-regulation of photosynthesis due to limited sink strength (Sheen, 1990; Stitt, 1991; Krapp *et al*., 1993; Moore *et al*., 1999; Paul and Pellny, 2003; Long *et al*., 2004) has been observed in elevated carbon dioxide concentration ([CO_2_]) experiments for C_3_ crops across various functional groups (e.g. Arp, 1991; Drake *et al*., 1997; Ainsworth *et al*., 2004; Ainsworth and Rogers, 2007; Leakey *et al*., 2009; Ruiz-Vera *et al*., 2017). Elevated [CO_2_] experiments are therefore a valuable platform to test sink limitations in plants, but such experiments have rarely been performed with cassava in field contexts.

Of the few studies examining cassava response to elevated [CO_2_] (Fernandez *et al*., 2002; Rosenthal *et al*., 2012; Cruz *et al*., 2014; Gleadow *et al*., 2009*a*; Forbes *et al*., 2020), only one (Rosenthal *et al*., 2012) grew cassava in the field under free-air CO_2_ enrichment (FACE). In that study, elevated [CO_2_] stimulated leaf photosynthesis (*A*, µmol CO_2_ m^−2^ s^−1^) by 30%, leading to a 104% and 90% increase in dry and fresh root biomass, respectively. However, that study used only one genotype (cv. 60444) not preferred by farmers in a truncated growing season, making it difficult to predict full season yield stimulations. Moreover, other cassava genotypes are likely to have considerable differences in sink capacity and phenology, which influence plant responses to elevated [CO_2_] (e.g. Pellet and El-Sharkawy, 1994; El-Sharkawy and De Tafur, 2007; Burns *et al*., 2010). Further investigation into cassava’s response to elevated [CO_2_] across multiple genotypes is therefore needed.

In addition to stimulating photosynthesis, elevated [CO_2_] also improves intrinsic water use efficiency (iWUE) in both C_3_ and C_4_ plants due to lower stomatal conductance (*g*_s_; Ainsworth and Long, 2005; Long *et al*., 2006; Ainsworth and Rogers, 2007; Bernacchi *et al*., 2007; Leakey *et al*., 2009). Both of these effects were detected in the previous cassava FACE experiment (Rosenthal *et al*., 2012) and can be beneficial under drought conditions, which are expected to be more frequent in sub-Saharan Africa (Rosenthal *et al*., 2012; Serdeczny *et al*., 2017).

Metabolite and nutrient contents also change under elevated [CO_2_] for many crops (Ainsworth and Long, 2005; Taub *et al*., 2008; McGrath and Lobell, 2013; Myers *et al*., 2014), often leading to decreased protein content in different plant tissues such as leaves (Ainsworth and Long 2005; Taub *et al*., 2008) and grains (Myers *et al*., 2014). Reduced leaf protein in cassava caused by increased atmospheric [CO_2_] would decrease its nutritional value. Reduced protein content could also alter cassava leaf toxicity because proteins help lower toxicity of cyanogenic glycoside compounds in cassava leaves (Gleadow *et al*., 2009*b*; Burns *et al*., 2010; McKey *et al*., 2010). Cassava produces the cyanogenic glycosides linamarin and lotaustralin (McMahon *et al*., 1995; Gleadow and Møller, 2014), which break down to release hydrogen cyanide (HCN) after mechanical disruption of the cells as a defense mechanism against herbivory (Conn, 1988; Gleadow and Woodrow, 2002; Gleadow and Møller, 2014). However, this also makes the tissue toxic for human consumption and can result in severe neurological diseases in humans (Mlingi *et al*., 1992; Nzwalo and Cliff, 2011). It is therefore very important to understand how nutritional quality and toxicity in cassava may be affected in plants grown at elevated [CO_2_].

In this study, we evaluated eight African farm-preferred cassava genotypes grown under elevated [CO_2_] using FACE technology in a 4 month field experiment to test for sink limitation. We hypothesized that cassava grown under elevated [CO_2_], independently of the cultivar, would not show indications of sink limitation after the formation of tuberous storage roots had started. As this was the first time that agronomically important cassava cultivars from Africa were grown under FACE conditions, we also conducted a general characterization of the effects of elevated [CO_2_] on the physiology, growth, biomass production, and toxicity of these eight cassava cultivars.

## Materials and methods

### Plant material, field site, and experimental design

The eight cultivars of cassava (*Manihot esculenta*) used in this study were obtained from the International Institute of Tropical Agriculture (IITA), Ibadan, Nigeria by the Swiss Federal Institute of Technology (ETH) (Zurich, Switzerland). They were: TME7, TMS98/0505, TME693, TMS98/0002, TMS01/1412, TME419, TMS30572, and TMS98/0581. This material was first inspected by the ETH for common viruses and bacteria and then sent to the University of Illinois at Urbana-Champaign (IL, USA) under an APHIS permit (permit number: PCIP-16-00268). The plantlets were propagated *in vitro* following Bull *et al*. (2009) and kept in a walk-in growth chamber at 28 °C, 16 h of light, and 50% relative humidity. Thirty-day-old plantlets were transferred to 9 cm diameter pots, kept for 2 weeks inside the greenhouse (at 28 °C, natural light, and ~60% relative humidity), and acclimated to the external environmental conditions for a week before being transplanted into the field.

The cassava FACE experiment (CassFACE) was performed in 2017 at the SoyFACE Global Change Research Facility (40.04N, 88.23W). CassFACE had eight heptagonal plots of 22 m diameter, four with elevated [CO_2_] (~600 μmol mol^−1^) and four with ambient [CO_2_] (~400 μmol mol^−1^). Plots were distributed in a randomized block design (*n*=4), and each block contained one ambient and one elevated [CO_2_] plot separated from each other by at least 100 m. The FACE system is described in more detail in Miglietta *et al*. (2001). Each cultivar was planted in subplots of 20 m^2^ within each plot. The location of the subplots within a plot was randomly distributed among blocks but maintained within a block. Cassava grew under FACE from 3 June to 30 September 2017 [day of the year (DOY) 154–273].

Before field transplantation, the soil was fertilized with 84 kg ha^−1^ of nitrogen. No herbicides or pesticides were applied. Transplanting was completed by block on DOY 154–156. In total, 35 plants were transplanted in each of the subplots with 20 as border plants. Plants were spaced at 0.7 m (between rows and plants within rows), and the subplots were spaced from each other by 1 m. Plants were hand-watered until the installation of a drip irrigation system (DOY 159), which maintained the equivalent of 25 mm of rainfall per week when precipitation was lacking. Air temperature was recorded every 10 min across the season at a local meteorological station. Daily precipitation was obtained from the University of Illinois Willard Airport weather station (40.04N, 88.28W) through the Midwestern Regional Climate Center (http://mrcc.isws.illinois.edu/CLIMATE/).

### Gas exchange measurements

Gas exchange measurements to determine *A* (µmol CO_2_ m^−2^ s^−1^), *g*_s_ (mol H_2_O m^−2^ s^−1^), and [CO_2_] inside the leaf (*C*_i_, µmol mol^−1^) were performed three times during the field season, between 12.45 h and 15.55 h on DOY 195, 10.45 h to 15.15 h on DOY 230, and 10.50 h to 13.50 h on DOY 269. These measurements were conducted using open gas exchange systems with an attached chlorophyll fluorometer chamber (LI-6400XT; LICOR, Inc., Lincoln, NE, USA). The gas exchange systems were calibrated as in Bernacchi *et al*. (2006).The photosynthetic photon flux density (PPFD; µmol m^−2^ s^−1^) and the chamber block temperature were set according to ambient conditions prior to the measurements. The values were: 27 °C and 1000 µmol m^−2^ s^−1^, 24 °C and 1650 µmol m^−2^ s^−1^, and 31 °C and 1900 µmol m^−2^ s^−1^ for the three respective days of measurement. The [CO_2_] inside the chamber was set to 400 µmol mol^−1^ or 600 µmol mol^−1^ depending on the [CO_2_] treatment. Relative humidity in the sample was maintained between 55% and 70%. The measurements were performed on the youngest fully expanded leaf of three cassava plants per subplot, after stabilization in the chamber for at least 3 min. Four gas exchange systems were used simultaneously, one in each block, with two measuring ambient and two measuring elevated [CO_2_] plots at any given time. *A*, *g*_s_, and *C*_i_ were calculated by the gas exchange system software following the equations of von Caemmerer and Farquhar (1981). iWUE (µmol mol^−1^) was calculated as *A*/*g*_s_.

Photosynthetic [CO_2_] response curves (*A/C*_i_ curves) were collected three times during the season (DOY 199–202, DOY 226–229, and DOY 267–269; see Supplementary Dataset S1 at *JXB* online) from the youngest fully expanded leaf of two plants per subplot. The [CO_2_] inside the chamber was varied as follows: 400, 300, 200, 100, 50, 400, 400, 600, 800, 1000, 1200, and 1500 µmol mol^−1^. PPFD was 1800 µmol m^−2^ s^−1^, leaf temperature was set to 30 °C for DOY 199–202 and to 28 °C for DOY 226–229 and DOY 267–269 (optimum temperature range for cassava growth; e.g. Pushpalatha and Gangadharan, 2020), and relative humidity in the sample chamber was ~70%. The ‘apparent’ maximum rate of carboxylation by Rubisco (apparent *V*_cmax_; µmol m^−2^ s^−1^) and ‘apparent’ maximum rate of photosynthetic electron transport (apparent *J*_max_; µmol m^−2^ s^−1^) were calculated at 28 °C using the equations from Farquhar *et al*. (1980) and Bernacchi *et al*. (2001, 2003). These values were designated ‘apparent’ because the calculations were based on *C*_i_ rather than the [CO_2_] inside the chloroplast (*C*_c_).

The J method (Harley *et al*., 1992) was used to calculate *g*_m_ (mol m^−2^ s^−1^) and *C*_c_ (µmol mol^−1^). The response of *A* to *C*_c_ (*A/C*_c_ curve) allowed the calculation of *V*_cmax_ and *J*_max_ following the equations in Harley *et al*. (1992) and the non-linear analysis with the Marquardt method from Moualeu-Ngangue *et al*. (2017). The Michaelis constant of Rubisco for CO_2_ (*K*_c_; µmol mol^−1^), the inhibition constant (*K*_o_; µmol mol^−1^), and the photorespiratory CO_2_ compensation point (Γ*; µmol mol^−1^) at the measured leaf temperature and at 25 °C were calculated using the scaling constant (*c*) and the enthalpies of activation (Δ*H*_a_) from Sharkey *et al*. (2007). *V*_cmax_ and *J*_max_ at 28 °C were obtained following the equations in Bernacchi *et al*. (2001, 2003). To calculate *g*_m_ at 28 °C, the *g*_m_ temperature response function required specific parameters for *c*, Δ*H*_a_, energies of deactivation (Δ*H*_d_), and entropy (Δ*S*), which were obtained from Bernacchi *et al*. (2002).

### Leaf area index, plant morphology characteristics, specific leaf area, and leaf carbon and nitrogen content

The leaf area index (LAI; m^2^ m^−2^) was recorded with an LAI-2200C plant canopy analyzer (LICOR, Inc.). Eleven measurements were obtained during the growing season (DOY 186, 193, 199, 209, 215, 222, 233, 248, 255, 262, and 272). In four plants per subplot, the following parameters were measured: plant height or main stem size (cm; DOY 220, 227, 235, 242, 252, 261, and 270), number of leaves on the main stem (DOY 165, 171, 178, 186, 193, 198, 206, 213, 220, 227, 242, 252, 261, and 270), the total number of leaves on the whole plant (DOY 235, 242, 252, 261, and 270), and the number of branches (DOY 242, 252, 261, and 270).

Specific leaf area (SLA; m^2^ kg^−1^), leaf carbon (C), and leaf nitrogen content (N; %, g m^−2^) were determined from leaf disks collected at midday twice during the season (DOY 195 and 230) from two plants per subplot. SLA was equal to the area of the disk divided by its dry weight. C and N content were quantified from 2 mg of fine powder sample using an elemental analyzer (Elemental Combustion System CHNS-O, Costech ECS 4010, Valencia, CA, USA). Leaf C:N was obtained by dividing % by weight C by % by weight N.

### Protein and non-structural carbohydrate content in leaves

Leaf disks of ~1.2 cm diameter were collected from two plants per subplot at dusk and at dawn on DOY 195–196, DOY 230–231, and DOY 269–270. Samples were immediately frozen in liquid nitrogen and stored at –80 °C until analysis. The amount of total soluble carbohydrates (TSCs; mmol hexose equivalents m^−2^) was quantified according to Ainsworth *et al*. (2007). First, HEPES-buffered ethanol (pH 7.8), 80% (v/v) and 50% (v/v), was heated to 80 °C and used to extract glucose, fructose, and sucrose from the leaves. Then, four enzymatic reactions (with hexokinase, phosphoglucose isomerase, glucose-6-phosphate dehydrogenase, and invertase) were performed for their quantification. The absorption of NADPH was measured at 340 nm after each reaction (Jones *et al*., 1977). Glucose in 70% ethanol was used as a standard. TSC was the sum of glucose+fructose+sucrose in the leaves but was expressed in glucose equivalents. After the soluble carbohydrates were extracted, the samples were ground and processed to obtain the amount of protein (g m^−2^) by using the Pierce™ BCA Protein Assay Kit (Cat No. 23227, Pierce, IL, USA). The protein content was determined spectrophotometrically at 562 nm using BSA as a standard.

For starch content (mg g^−1^), 10 mg of freeze-dried samples was ground to a fine powder and washed six times with 80% ethanol at 80 °C. Starch was extracted from the remaining material after washes with enzymatic reactions using α-amylase (120 U ml^−1^) and amyloglucosidase (30 U ml^−1^). Glucose released from enzymatic reactions was quantified by spectrophotometry at 490 nm after the reaction with an oxidase/peroxidase assay kit (NZYtech, Lisboa, Portugal) (De Souza *et al*., 2013; De Souza and Long, 2018). Glucose was used as a standard. The hydrolysis of starch to glucose requires one molecule of water in each covalent bond hydrolyzed; thus, the amount of starch was equivalent to 90% of the total glucose released after extraction (Amaral *et al*., 2007).

The rate of turnover of TSCs and starch (i.e. the use of carbohydrate during the night) was calculated by subtracting the amount of carbohydrates (TSCs or starch) obtained at dusk from the amount obtained from the following dawn.

### Determination of hydrogen cyanide content in leaves and roots

The amount of cyanogenic glucosides was measured in leaves and the outer and inner tissues of the storage roots by measuring the amount of HCN evolved from the tissues. Leaf samples were collected at midday from three plants per subplot during DOY 195, 230, and 269. Root samples, peel (periderm tissue), and core (starchy parenchyma) were extracted from three plants per subplot during the final harvest. After collection, samples were immediately frozen in liquid nitrogen and stored at –80 °C and then freeze-dried for analysis. The protocol to determine HCN (mg g^−1^ of dry mass) was from Gleadow *et al*. (2016). In summary, 5 mg of freeze-dried and ground tissue was transferred to vials containing 300 µl of 0.1 M phosphate buffer (pH 6.4) and latex (100:1 v/v) collected from cassava plants that contained the β-glucosidase required to degrade the cyanogenic glucosides to HCN. Microtubes (0.2 ml) containing 200 µl of 1 M NaOH were inserted into the vials. The vials were sealed and then frozen and rethawed to room temperature twice to disrupt the cells and ensure mixing of cyanogenic glucosides with the degradative enzymes, and then incubated for 19 h at 37 °C. Volatile HCN from the samples was trapped in the NaOH in the inner 0.2 ml microtubes. The concentration of HCN in the NaOH was determined colorimetrically. The absorbance was determined at 595 nm using sodium cyanide (NaCN) as a standard. The HCN content was calculated in mg g^−1^ of dry mass.

### Fresh weight, dry weight, and harvest index

The final harvest, DOY 275–286, was conducted block by block to ensure that plants from ambient and elevated [CO_2_] were harvested at a similar time. Fresh weights of the above-ground biomass (AGB; 15 plants per subplot) and roots (9 per subplot) were recorded. The dry weights of AGB and roots were obtained from five plants dried at 60 °C until constant weight. The harvest index (HI) in fresh and dry biomass was equal to the weight of roots divided by weight of AGB+roots.

### Statistical analysis

This experiment was a split-split-plot design in which variables were analyzed with a mixed model ANOVA (PROC MIXED, SAS System 9.4, SAS Institute, Cary, NC, USA). Repeated measurements were applied when data from a variable were collected more than once during the growing season. DOY was the repeated measurement factor for the seasonal analysis. The fixed effects were [CO_2_], cultivar, DOY, and their interactions. Block was a random effect. The Kenward–Roger method was used to calculate the degrees of freedom. Pairwise comparisons were performed by the least square means test (*t*-test) with significance determined as *P*-value ≤0.1.

The contribution of each variable for the differences observed between treatments and cultivars was evaluated with a principal component analysis (PCA). PCA was performed using the data presented in the main figures of this manuscript (JMP^®^Pro, version 12.0.1; SAS Institute) to check, among the variables most relevant for the discussion of this dataset, which ones would better explain the differences between treatments and among cultivars. For LAI, height, number of leaves on the main stem and on the whole plant, and number of branches, the PCA considered data from the final measurements of the season (from DOY 270 to 272). For all other variables, PCA was performed using the seasonal averages. Since no significant differences were observed in protein content between dusk and dawn, data from dawn were used for the PCA to avoid redundancy of values for this parameter. The data were normalized using log10 function. To avoid negative values in the PCA matrix (e.g. TSC turnover), a constant value was added to the data prior to the log transformation.

## Results

### Meteorological conditions

**Fig. 1.**
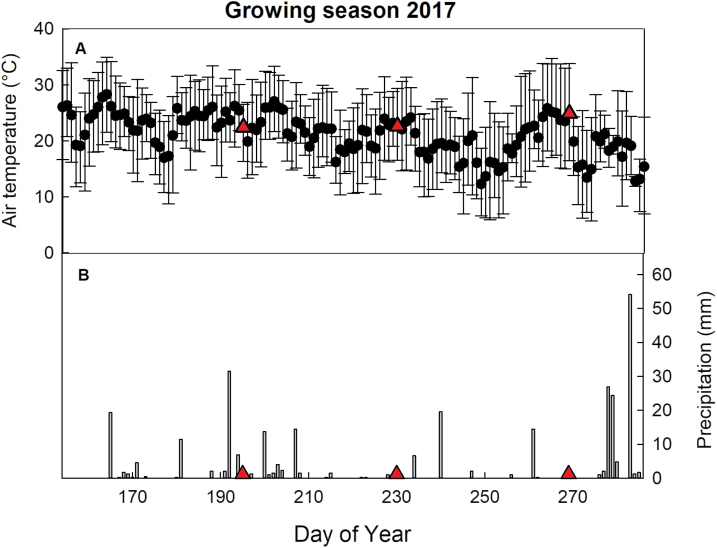
Daily mean air temperature (°C, black circles, A) with maximum and minimum values (top and bottom of the error bars), and daily precipitation (mm, gray bars, B) for the 2017 cassava growing season (from planting to the end of the final harvest: DOY 154–286). Drip irrigation was used when precipitation rates fell below 25 mm per week. Red triangles indicate the days when leaf carbon assimilation measurements and midday samplings were taken (DOY 195, DOY 230, and DOY 269).

The 2017 growing season (DOY 154–286; Fig. 1) received ~40% less rainfall than the average annual rainfall for the Champaign-Urbana area over the 20 year period from 1996 to 2016 (Midwestern Regional Climate Center). Drip irrigation augmented rainfall such that plots received the equivalent of ~25 mm of rainfall per week, a value within the range of rainfall received in a growing season in Nigeria (~1500 mm year^–1^, Ajetomobi, 2016). The monthly mean temperatures were 23.1, 23.6, 20.3, and 19.2 °C for June, July, August, and September, respectively (Fig. 1). The minimum daily temperatures during these months ranged from 5.7 °C to 13.3 °C, and the maximum daily temperatures ranged from 28.9 °C to 34.9 °C (Fig. 1).

### Gas exchange parameters differed between ambient and elevated [CO_2_] treatments

**Fig. 2.**
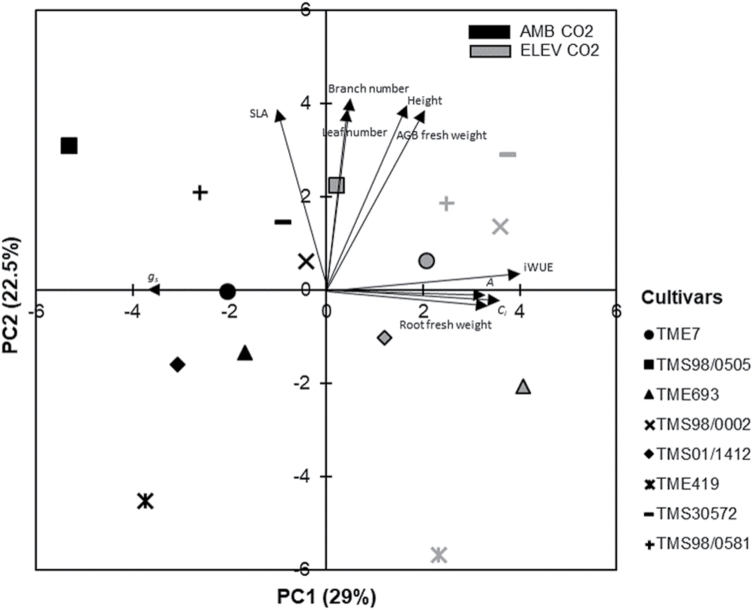
Principal component analysis (PCA) of data collected from eight cassava cultivars grown at ambient (AMB CO_2_) and elevated [CO_2_] (ELE CO_2_). The percentage on each axis represents the contribution of each principal component for the differences observed between [CO_2_] treatments (*x*-axis) and between cultivars (*y*-axis). Only the arrows representing the variables that most explained the variation in this experimental dataset are labeled in this figure. The list of all variables used in the PCA with the respective eigenvalues is shown in Supplementary Table S1.

The elevated [CO_2_] treatment comprised 29% of the total variation observed in the data of this experiment (Fig. 2). The enhancement of *A*, *C*_i_, and iWUE and the reduction of *g*_s_ under elevated [CO_2_] were among the main contributors of all measured variables to the differences between ambient- and elevated [CO_2_]-grown plants (Fig. 2; Supplementary Table S1).

**Fig. 3.**
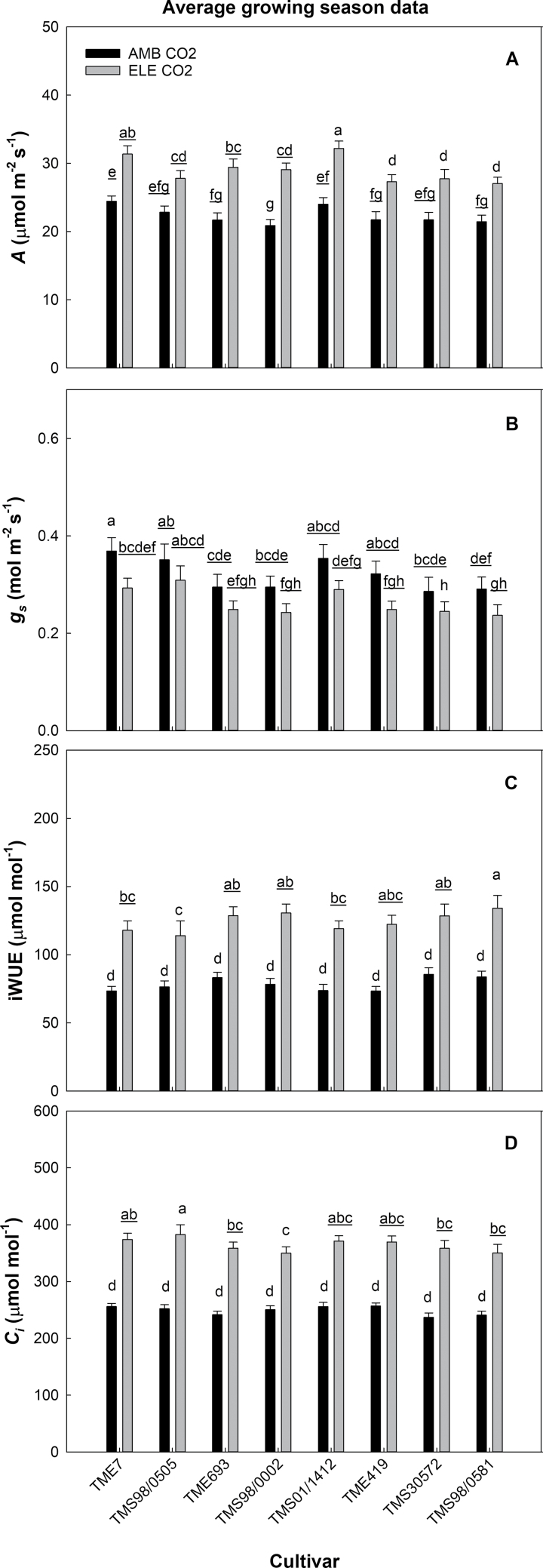
Seasonal average of photosynthetic carbon uptake (*A*, µmol CO_2_ m^−2^ s^−1^, A), stomatal conductance (*g*_s_, mol H_2_O m^−2^ s^−1^, B), intrinsic water use efficiency (iWUE, µmol mol^−1^, C), and [CO_2_] inside the leaf (*C*_i_, µmol mol^−1^, D) from eight cultivars of cassava grown at ambient (AMB CO_2_) and elevated [CO_2_] (ELE CO_2_). Values are means ±SE (*n*=4). Treatments with different letters represent significant differences (*P*<0.1); underlining is used to help differentiate groups of letters.

Elevated [CO_2_] increased *A* by 31% (+7 µmol of CO_2_ m^−2^ s^−1^) averaged across all cultivars over the entire growing season (Fig. 3; Table 1; Supplementary Fig. S1; Supplementary Table S2), but the magnitude of that increase differed among cultivars (Table 1; Supplementary Table S2). TMS98/0002 exhibited the largest *A* response to elevated [CO_2_] (+41%), while TMS98/0505 exhibited the smallest (+23%; Fig. 3). The CO_2_ effect on *A* increased throughout the season for all cultivars (Table 1), such that *A* increased on average 18, 34, and 41% on DOY 195, 230, and 269, respectively (Supplementary Fig. S1; Supplementary Table S2).

**Table 1.** Statistical analysis (ANOVA) for seasonal averages or for the final harvest data

Type of measurements	Parameters	Main effects						
		[CO_2_]	Cultivar	[CO_2_]× cultivar	DOY	DOY× [CO_2_]	DOY×cultivar	DOY×[CO_2_]×cultivar
Gas exchange	*A*	<0.0001	<0.001	ns	<0.0001	0.030	ns	ns
	*g*_s_	<0.0001	0.006	ns	<0.0001	0.013	ns	ns
	iWUE	0.003	ns	ns	<0.0001	<0.001	0.007	ns
	*C*_i_	<0.0001	ns	ns	<0.0001	<0.0001	0.007	ns
	Apparent *V*_*cmax*_	ns	ns	ns	<0.0001	ns	ns	ns
	Apparent *J*_max_	ns	ns	ns	<0.0001	ns	ns	ns
	g_m_	ns	ns	0.046	<0.0001	0.086	ns	ns
	*V*_cmax_	ns	0.039	ns	<0.0001	ns	ns	ns
	*J*_max_	ns	ns	ns	0.001	0.082	ns	ns
Plant growth	LAI	<0.0001	<0.0001	0.003	<0.0001	0.003	<0.0001	ns
	Height	0.082	<0.0001	<0.0001	<0.0001	ns	<0.0001	ns
	No. of leaves on main stem	ns	<0.0001	<0.001	<0.0001	ns	<0.0001	ns
	No. of leaves in the whole plant	0.001	<0.0001	<0.001	<0.0001	ns	<0.0001	ns
	No. of branches	ns	<0.0001	<0.0001	<0.0001	ns	<0.0001	ns
	SLA	0.049	<0.0001	ns	<0.0001	ns	0.073	ns
Plant material composition	Leaf N	ns	<0.0001	ns	<0.0001	0.009	0.013	ns
	C:N	0.009	<0.0001	ns	<0.0001	0.009	<0.0001	ns
	Protein dusk	ns	<0.0001	ns	<0.0001	ns	0.027	ns
	Protein dawn	ns	<0.0001	ns	<0.001	ns	0.005	ns
	TSC dusk	0.006	<0.001	<0.0001	<0.0001	ns	ns	ns
	TSC dawn	0.008	<0.0001	ns	<0.0001	ns	<0.0001	0.034
	Starch dusk	<0.0001	<0.0001	0.074	<0.0001	0.019	0.001	ns
	Starch dawn	<0.0001	<0.0001	ns	<0.0001	ns	0.046	ns
	TSC turnover	ns	<0.001	0.040	<0.0001	ns	ns	0.058
	Starch turnover	ns	ns	ns	ns	<0.001	ns	ns
	HCN in leaves	0.028	ns	ns	<0.0001	0.031	ns	ns
	HCN in peel of roots	ns	0.006	ns	–	–	–	–
	HCN in core of roots	ns	<0.0001	ns	–	–	–	–
Biomass and yield	Ffresh weight of roots	0.002	<0.0001	ns	–	–	–	–
	Fresh weight of AGB	0.008	<0.0001	ns	–	–	–	–
	Fresh weight HI	ns	<0.0001	ns	–	–	–	–
	Dry weight of roots	0.005	<0.0001	ns	–	–	–	–
	Dry weight of AGB	0.005	ns	ns	–	–	–	–
	Dry weight HI	ns	<0.0001	ns	–	–	–	–

The following parameters were measured: photosynthetic carbon uptake (*A*, µmol CO_2_ m^−2^ s^−1^), stomatal conductance (*g*_s_, mol H_2_O m^−2^ s^−1^), intrinsic water use efficiency (iWUE, µmol mol^−1^), [CO_2_] inside the leaf (*C*_i_, µmol mol^−1^), ‘apparent’ maximum rate of carboxylation by Rubisco (apparent *V*_cmax_, µmol m^−2^ s^−1^), ‘apparent’ maximum rate of photosynthetic electron transport (apparent *J*_max_, µmol m^−2^ s^−1^), mesophyll conductance (*g*_m_, mol m^−2^ s^−1^), *V*_cmax_, *J*_max_, leaf area index (LAI, m^2^ m^−2^), height (cm), number of leaves on the main stem and in the whole plant, number of branches, specific leaf area (SLA, m^2^ kg^−1^), leaf nitrogen (g m^−2^), carbon versus nitrogen ratio (C:N), protein content (dusk and dawn; g m^−2^), total soluble carbohydrates (TSC, mmol m^−2^) and starch (mg g^−1^) at dusk and dawn, TSC and starch turnover, hydrogen cyanide (HCN) content in leaves and roots (mg g^−1^), fresh and dry weight of roots (g plant^−1^), above-ground biomass (AGB, g plant^−1^), and harvest index (HI)

The main effects are: [CO_2_], cultivar, day of the year (DOY), and their interaction. Significant (*P*<0.1) and non-significant (ns) differences are shown in the table.

CO_2_ also significantly impacted *g*_s_, but the magnitude varied by cultivar and DOY (Table 1). Elevated [CO_2_] reduced season averages of *g*_s_ by 16% across all cultivars, ranging from a 12.5% reduction in TMS30572 to a 20% reduction in TME7 (Fig. 3). Despite the strong CO_2_ effect on *g*_s_ observed in TME7, this cultivar, together with the cultivars TMS98/0505 and TMS01/1412, showed the highest *g*_s_ at both [CO_2_] levels (Fig. 3; Supplementary Fig. S1; Supplementary Table S2).

iWUE and *C*_i_ increased under elevated [CO_2_] on all dates (Supplementary Fig. S1; Supplementary Table S2), with a seasonal average increase of 58% in iWUE and 46% in *C*_i_ (Table 1). While the values of iWUE and *C*_i_ were mostly similar across the cultivars under ambient [CO_2_], the extent of their increase under elevated [CO_2_] differed significantly among cultivars (Fig. 3; Supplementary Fig. S1). The largest variation was observed on DOY 230, when the increase in iWUE ranged from 19% (not significantly different) in TMS98/0505 to 73% in TMS98/0002 (Supplementary Fig. S1). That same day, the increase in *C*_i_ under elevated [CO_2_] was 38% in TMS98/0002 and 70% in TMS98/0581 (Supplementary Fig. S1).

### *V*_cmax_, *J*_max_, and *g*_m_ were not affected by elevated [CO_2_]

**Fig. 4.**
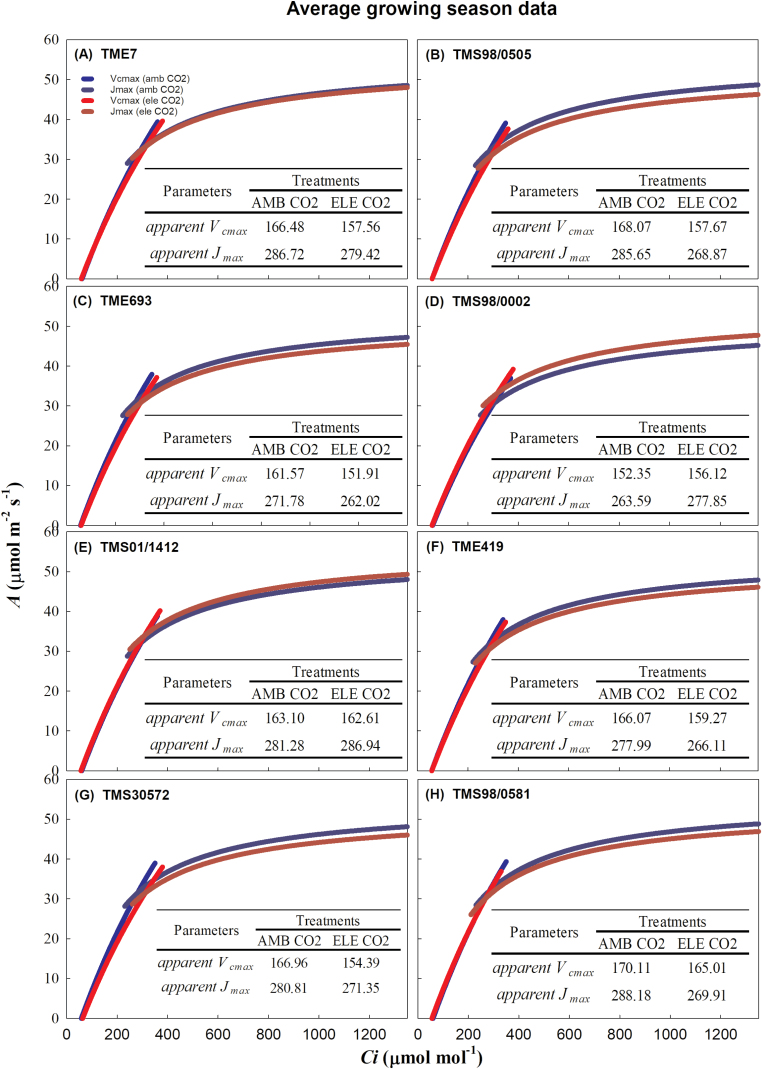
Fitted responses of *A*/*C*_i_ curves at 28 °C. The seasonal average of the apparent *V*_cmax_ (µmol m^−2^ s^−1^, SE <3.6–8.3>) and apparent *J*_max_ (µmol m^−2^ s^−1^, SE <7.2–14.9>) from eight cultivars of cassava (from A–H) grown at ambient (AMB CO_2_; blue lines) and elevated (ELE CO_2_; red lines) [CO_2_] are indicated in the inserted tables. Effects of elevated [CO_2_] on apparent *V*_cmax_ and apparent *J*_max_ were not significant. The blue and red vertical lines represent the supply functions (1/–*g*_s_) for the ambient and elevated [CO_2_] treatments, respectively. The supply functions intercept the fitted *A*/*C*_i_ at the operating point.

**Fig. 5.**
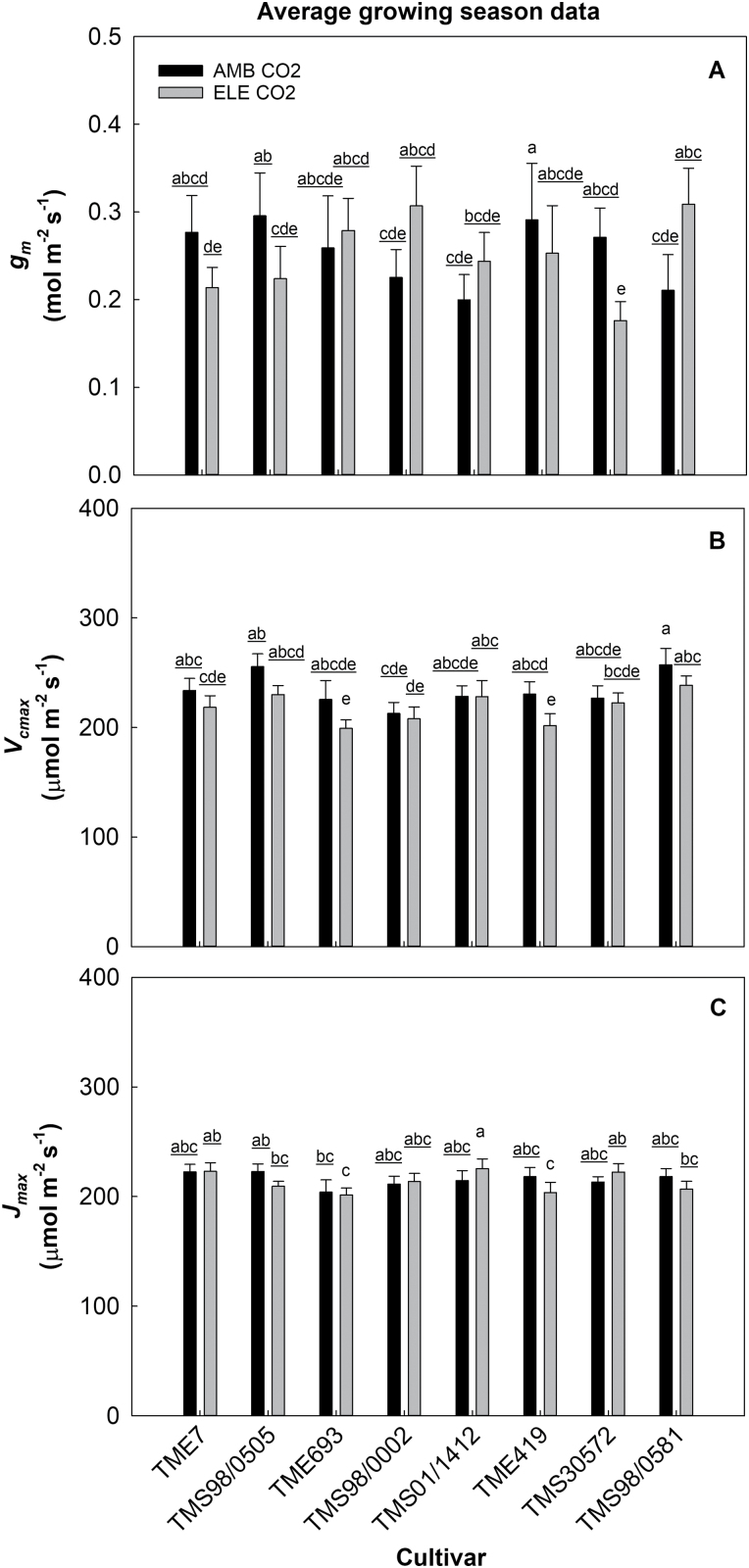
Seasonal averages of mesophyll conductance (*g*_m_, mol m^−2^ s^−1^, A), the maximum carboxylation rate by Rubisco (*V*_cmax_, µmol m^−2^ s^−1^ B), and the regeneration of ribulose-1,5-bisphosphate controlled by the electron transport rate (*J*_max_, µmol m^−2^ s^−1^ C) at 28 °C from eight cultivars of cassava grown at ambient (AMB CO_2_) and elevated [CO_2_] (ELE CO_2_). Values are means ±SE (*n*=4). Treatments with different letters represent significant differences (*P*<0.1); underlining is used to help differentiate groups of letters.

When averaged over the growing season, elevated [CO_2_] did not affect the apparent *V*_cmax_, apparent *J*_max_, *V*_cmax_, or *J*_max_ in any of the cultivars (Figs 4, 5; Table 1; Supplementary Figs S2–S5). However, the analysis per DOY showed that during the first set of measurements (DOY 199–202) the apparent *V*_cmax_ declined under elevated [CO_2_] in three cultivars (TME7, TMS98/0505, and TMS98/0581) by ~7% (Supplementary Fig. S2; Supplementary Table S2). Concurrently, the apparent *J*_max_ declined by 9% in TMS98/0581 and increased by 17% in TMS01/1412 (Supplementary Fig. S2; Supplementary Table S2). These parameters varied among cultivars by the end of the experiment (DOY 267–269; Supplementary Fig. S4; Supplementary Table S2). Similarly, *V*_cmax_ varied across cultivars; this difference was significant during DOY 226–229 and DOY 267–269 (Supplementary Fig. S5; Supplementary Table S2). TMS98/0581, TMS98/0505, and TMS01/1412 had the highest values of apparent *V*_cmax_, apparent *J*_max_, and *V*_cmax_ independent of [CO_2_] treatment during the third set of measurements (DOY 267–269), whereas TMS693 had the lowest values (Supplementary Figs S4, S5).

Elevated [CO_2_] only affected *g*_m_ in TMS30572 and TMS98/0505, in which *g*_m_ declined at elevated [CO_2_] on average by ~35% across the season (Table 1; Fig. 5). *g*_m_ varied among cultivars only at the beginning of the season (DOY 199–202; Supplementary Fig. S5; Supplementary Table S2). The overall lack of a significant effect of elevated [CO_2_] on *g*_m_ contributed to the similar CO_2_ responses for the apparent *V*_cmax_/apparent *J*_max_, and these same parameters calculated using *g*_m_ (*V*_cmax_/*J*_max_) (Figs 4, 5; Table 1; Supplementary Figs S2–S5; Supplementary Table S2).

### Elevated [CO_2_] stimulation of cassava growth parameters varied across cultivars

**Fig. 6.**
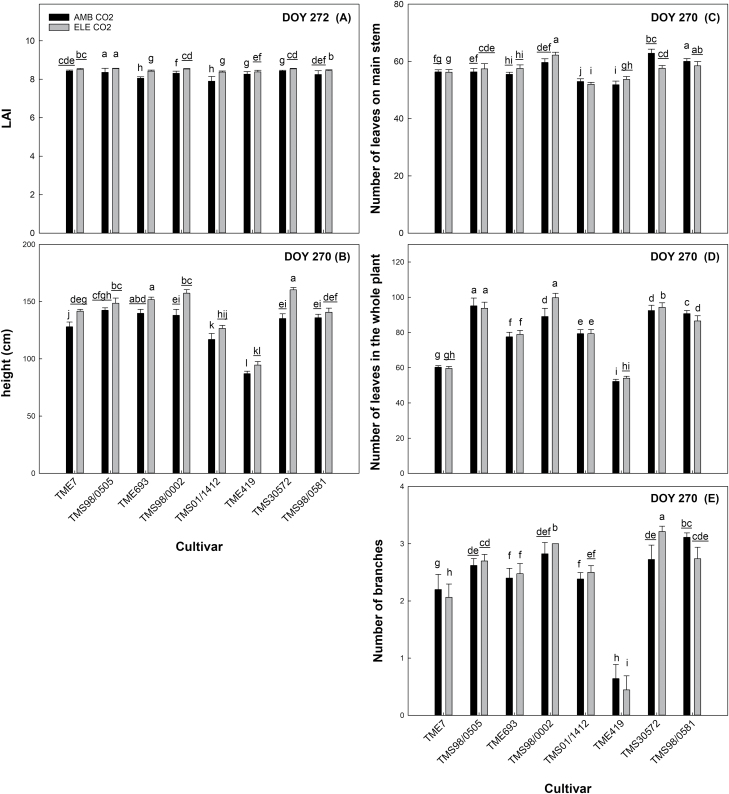
Daily average of growth parameters on the last day of measurements. Leaf area index (LAI; m^2^ m^−2^, A), height (cm, B), number of leaves on the main stem (C) and whole plant (D), and number of branches (E) from eight cultivars of cassava grown at ambient (AMB CO_2_) and elevated [CO_2_] (ELE CO_2_). Values are means ±SE (*n*=4). Treatments with different letters represent significant differences (*P*<0.1) for the seasonal results; underlining is used to help differentiate groups of letters. See Supplementary Fig. S6 for all daily measurements.

Growth parameters comprised the majority of the variation (PC2; 22.5%) found among cultivars (Fig. 2; Supplementary Table S1). In addition, growth parameters also contributed to the variation observed between ambient and elevated [CO_2_], showing significant differences between these two treatments (Table 1). Elevated [CO_2_] increased LAI (Fig. 6; Supplementary Fig. S6), which was the growth variable that most contributed to the differences between ambient and elevated [CO_2_] (higher value for PC1; Supplementary Table S1). During the season, LAI increased by 6% to 17% in elevated [CO_2_] as compared with ambient [CO_2_] in six of the eight cultivars (TME693, TMS98/0002, TMS01/1412, TME419, TMS30572, TMS98/0581; Supplementary Fig. S6). However, the difference between [CO_2_] treatments decreased over the growing season and, by the end, this increase was only ~3% (Fig. 6; Supplementary Fig. S6). Throughout the season, TMS98/0505 maintained the largest LAI while TMS01/1412 and TME693 had the smallest (Supplementary Fig. S6).

Plant height increased at elevated [CO_2_] with a statistically significant 12–20% increase in height in four cultivars (TME7, TMS98/0002, TMS01/1412, and TMS30572; Fig. 6; Table 1; Supplementary Fig. S6). By the end of the growing season, the tallest cassava cultivar was TMS30572 grown at elevated [CO_2_] (~160 cm), and the shortest was TME419 grown at ambient [CO_2_] (~95cm) (Fig. 6). The number of leaves in cassava (on the main stem and on the whole plant) increased under elevated [CO_2_] conditions (Table 1) but depended on cultivar and measurement day. By the end of the experiment, the total number of leaves on the whole plant ranged from 50 to 100 leaves at either ambient or elevated [CO_2_], depending on the cultivar (Fig. 6). Branching ranged from almost no branches to many branches depending on the cultivar (Table 1), with the fewest branches in TME419 and TME7 (Fig. 6). Elevated [CO_2_] decreased the number of branches in these two cultivars without changes in leaf number, whereas branch number increased in TMS98/0002 and TMS30572 (Fig. 6; Table 1; Supplementary Fig. S6).

**Fig. 7.**
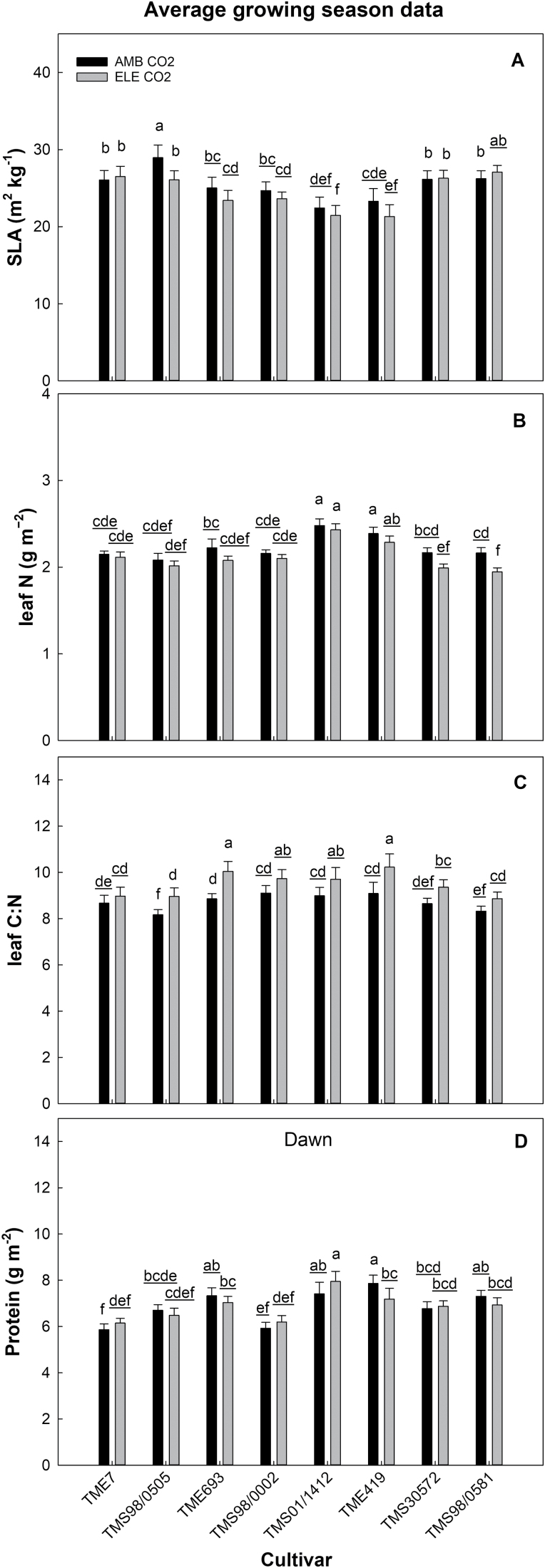
Seasonal average of the specific leaf area (SLA; m^2^ kg^−1^, A), leaf nitrogen (g m^−2^, B), carbon and nitrogen ratio (C:N, C), and protein content (at dawn; g m^−2^, D) from eight cultivars of cassava grown at ambient (AMB CO_2_) and elevated [CO_2_] (ELE CO_2_). Values are means ±SE (*n*=4). Treatments with different letters represent significant differences (*P*<0.1); underlining is used to help differentiate groups of letters.

Overall, SLA tended to be lower at elevated [CO_2_] (Table 1); however, this reduction was significant only in TMS98/0505, which had the highest SLA at ambient [CO_2_] (average of 28.95 m^2^ kg^−1^) with a reduction of ~9% at elevated [CO_2_] for the season (Fig. 7; Supplementary Fig. S7).

### Leaf N and protein content were not altered under elevated [CO_2_]

In general, the seasonal (DOY) and daily (at either dusk or dawn) analysis showed no changes in the leaf N or protein content on a leaf area basis with elevated [CO_2_] (Fig. 7; Table 1; Supplementary Figs S7, S8; Supplementary Table S3), but there were differences among cultivars. TMS01/1412 and TME419 had the highest leaf N and protein content across all the measurements (Supplementary Figs S7, S8).

The C:N ratio increased by 8% under elevated [CO_2_] across all days (Fig. 7; Table 1; Supplementary Table S3); this was driven by increased C because leaf N did not change. Regardless of the treatment and cultivar, C:N increased as the season progressed, with a 28% higher C:N on DOY 230 than on DOY 195 (Supplementary Fig. S7).

### Elevated [CO_2_] increased leaf carbohydrates in cassava

**Fig. 8.**
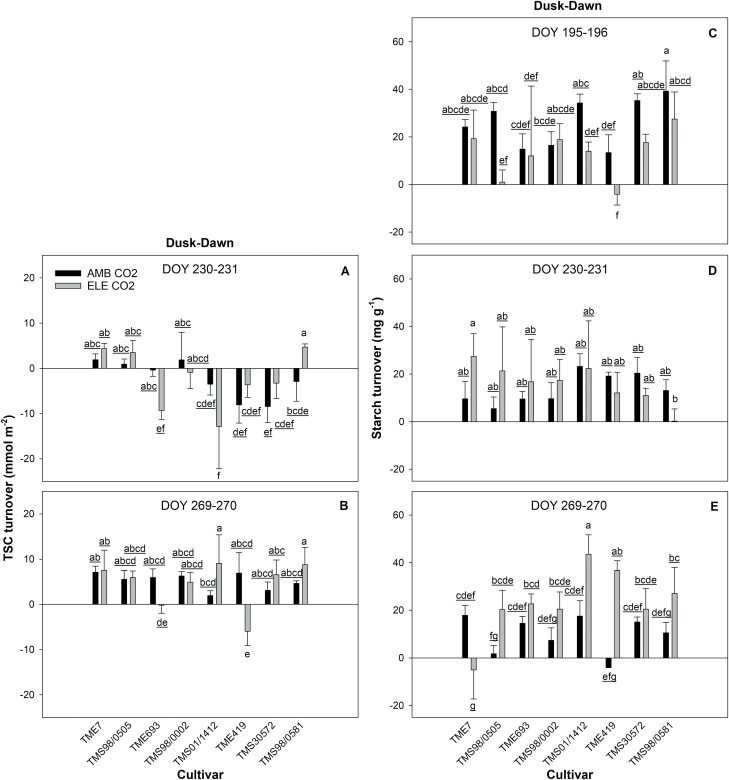
Daily average of the turnover of total soluble carbohydrates (TSC; mmol m^−2^, A, B) and starch turnover (mg g^−1^, C–E) from eight cultivars of cassava grown at ambient (AMB CO_2_) and elevated [CO_2_] (ELE CO_2_). Values are means ±SE (*n*=4). Treatments with different letters represent significant differences (*P*<0.1); underlining is used to help differentiate groups of letters.

Elevated [CO_2_] increased TSC by 19–39% at either dusk or dawn (Table 1; Supplementary Fig. S9; Table S3) in all the cultivars (except for TME419) on at least one of the days of measurement (DOY 230–231 and DOY 269–270). TSC turnover indicates the rate at which TSC is used by the plant during the night and was not affected by the [CO_2_] treatment alone (Fig. 8; Table 1; Supplementary Table S3). However, TSC turnover varied depending on the cultivar and its interaction with CO_2_ (Table 1; Supplementary Table S3). In the middle of the season (DOY 230–231), the use of TSC during the night was low (negative values) for TME693, TMS01/1412, TME419, and TMS30572 regardless of [CO_2_] (Fig. 8; Supplementary Fig. S9). The higher amount of glucose at dawn than at dusk for these cultivars (Supplementary Fig. S9) may occur if another pool of carbohydrates has been used to supply more glucose (e.g. starch). With the exception of TME419 at elevated [CO_2_], the rate of turnover increased for TSC significantly during the season for all cultivars (Fig. 8; Supplementary Fig. S9).

Starch content increased when plants were grown under elevated [CO_2_] at dusk and dawn on all the measurement days (Table 1; Supplementary Fig. S10; Supplementary Table S3). Depending on the cultivar, the increase in starch ranged from 38% to 508% at dusk (in TMS01/1412 and TMS98/0505, respectively) and from 30% to 540% at dawn (in TME693 and TMS30572, respectively) (Supplementary Fig. S10). TMS98/0002 did not show a significant alteration in starch content at dawn under elevated [CO_2_]. Starch content was higher earlier in the season (DOY 195–196) as compared with later in the season (DOY 230–231 and DOY 269–270; Table 1; Supplementary Fig. S10). Starch turnover showed a significant CO_2_ effect on two of the three sampling days (DOY 195–196 and DOY 269–270; Fig. 8; Table 1; Supplementary Table S3). Interestingly, starch turnover was ~50% lower at elevated [CO_2_] than at ambient [CO_2_] during the first set of measurements (Fig. 8). However, starch turnover under elevated [CO_2_] accelerated over the season, and was >150% higher than at ambient [CO_2_] by the end of experiment (Fig. 8; Supplementary Fig. S10).

### Elevated [CO_2_] decreased HCN content in leaves but not in the storage roots

**Fig. 9.**
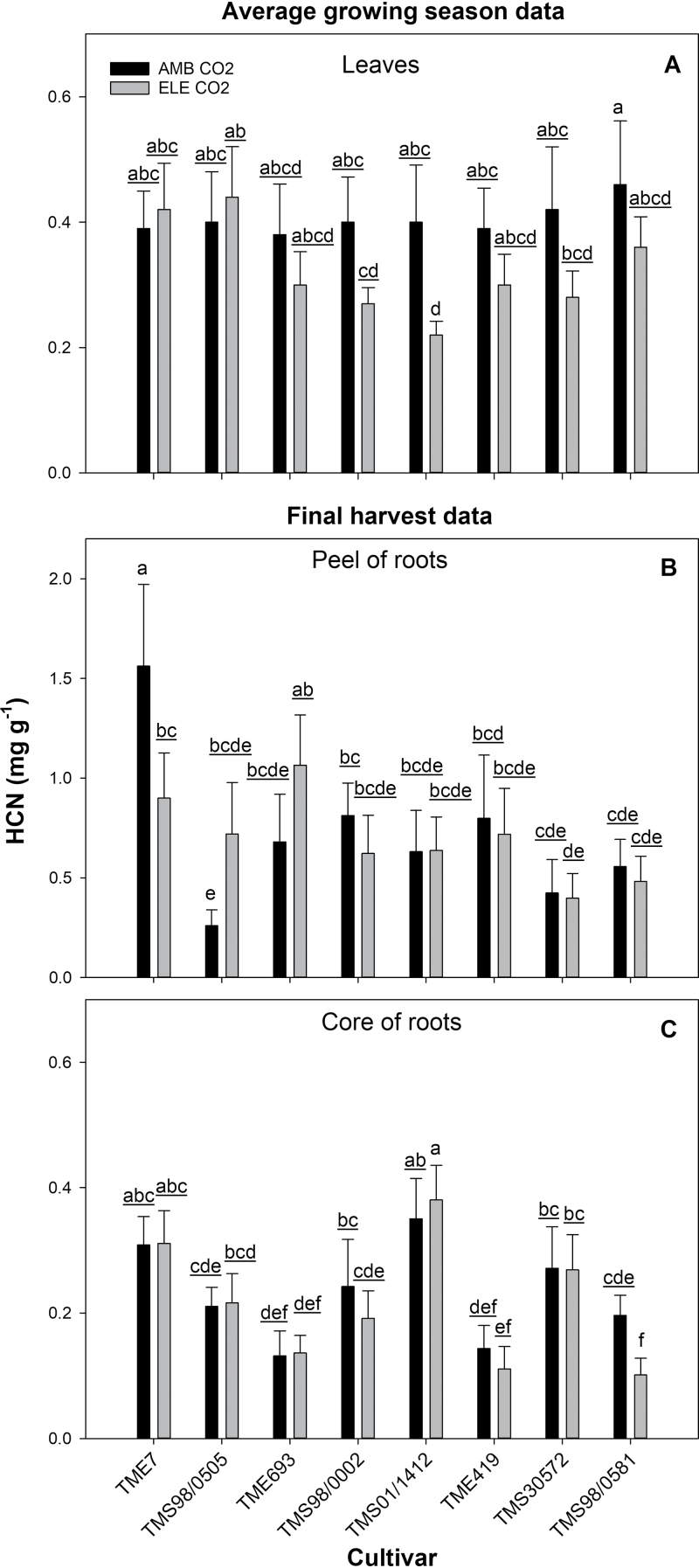
Hydrogen cyanide content (mg g^−1^) in leaves (A) and root tissues (B and C) of eight cultivars of cassava grown at ambient (AMB CO_2_) and elevated [CO_2_] (ELE CO_2_). Values are means ±SE (*n*=4). Treatments with different letters represent significant differences (*P*<0.1); underlining is used to help differentiate groups of letters.

Despite differences in HCN among cultivars, elevated [CO_2_] did not significantly affect HCN content in the peel or core of the storage roots on a dry weight basis (Table 1; Fig. 9). In the leaves, however, elevated [CO_2_] generally reduced HCN on a dry weight basis in TMS98/0002 (38% reduction on DOY 230) and TMS01/1412 (60% reduction on DOY 269) (Fig. 9; Supplementary Fig. S11; Supplementary Table S3). An increase in HCN content with elevated [CO_2_] only occurred in TMS98/0505 (DOY 230; Supplementary Fig. S11).

### Elevated [CO_2_] increased root biomass and AGB but not HI

**Fig. 10.**
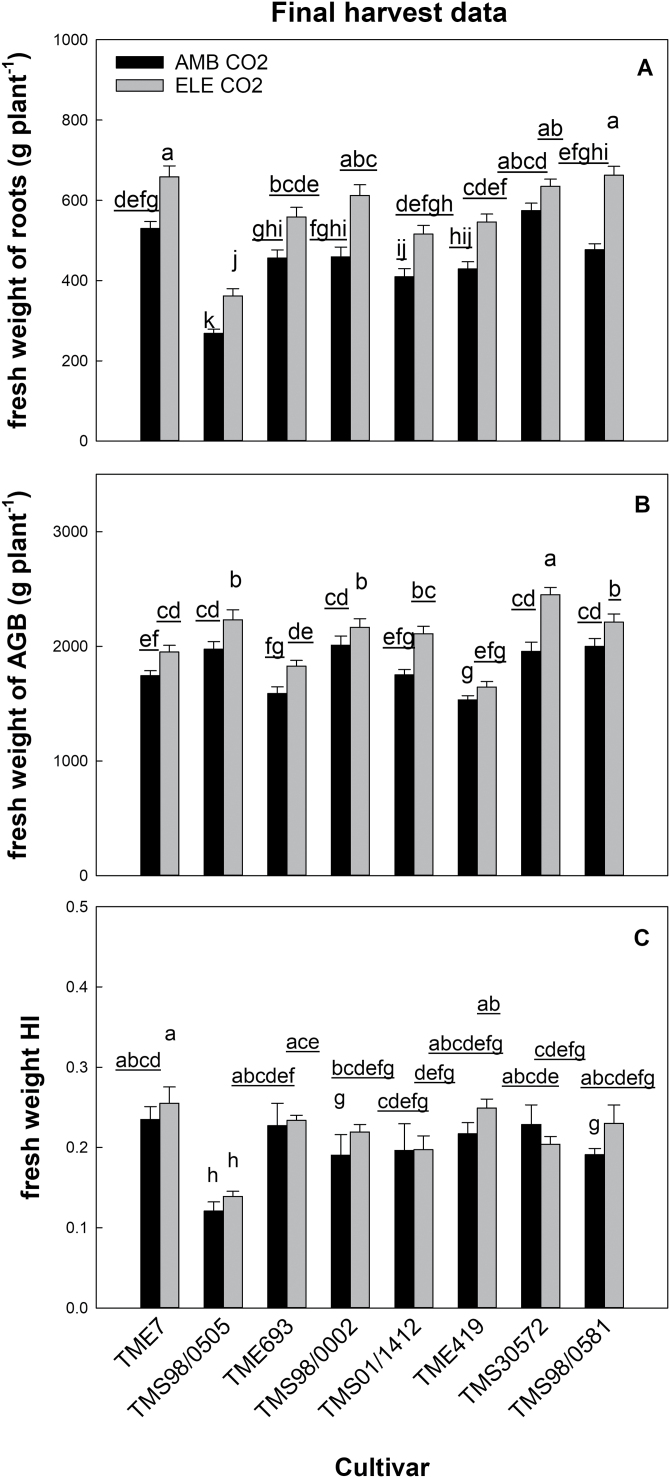
Average fresh weight of roots (g per plant, A), and above-ground biomass (AGB; g per plant, B), and harvest index (HI, C) from eight cultivars of cassava grown at ambient (AMB CO_2_) and elevated [CO_2_] (ELE CO_2_). Values are means ±SE (*n*=4). Treatments with different letters represent significant differences (*P*<0.1); underlining is used to help differentiate groups of letters.

Average fresh weight of roots across cultivars increased by 27% under elevated [CO_2_] and, together with the gas exchange parameters, was one of the main contributors to the differences in parameter responses between ambient and elevated [CO_2_] growth conditions (Fig. 2; Supplementary Table S1). This stimulation occurred in all cultivars with the exception of TMS30572 (Fig. 10; Table 1). The highest elevated [CO_2_] stimulation occurred in TMS98/0581 and TMS98/0505 with 39% and 35% increases in root fresh biomass, respectively. However, TMS98/0505 had the lowest root biomass by the end of the experiment compared with the other cultivars at both CO_2_ levels (Fig. 10). On a dry weight basis, root biomass had statistically significant increases of ~37% at elevated [CO_2_] in four cultivars (TME7, TMS98/0505, TMS98/0002, and TMS98/0581; Supplementary Fig. S12). Although not significant, the percentage increase in dry root biomass at elevated [CO_2_] in the other four cultivars ranged from 5% to 20% compared with ambient. Root biomass (fresh and dry weight) expressed in t ha^−1^ (yield units) is shown in Supplementary Fig. S13.

Fresh and dry weight of AGB also increased under elevated [CO_2_] (Fig. 10; Table 1; Supplementary Fig. S12), ranging from 8% in TMS98/0002 to 25% in TMS30572. Only TME419 did not show a significant increase in fresh weight of AGB at elevated [CO_2_] (Fig. 10). Fresh weight of AGB also differed among the cultivars, with the lowest AGB in TME419 (mean of 1590 g per plant independently of the [CO_2_] treatment) and the highest in TMS30572 (~2450 g per plant at elevated [CO_2_]; Fig. 10). Dry weight of AGB showed an overall increase with elevated [CO_2_] but this was statistically significant only in TMS30572 (37%; Supplementary Fig. S12). Elevated [CO_2_] had no significant effects on HI (Table 1). Among the cultivars and regardless of the [CO_2_] treatment, the highest HI after 4 months of growth was from TME7 (0.24 for fresh and 0.39 for dry weight HI) and the lowest was from TMS98/0505 (0.13 for fresh and 0.23 for dry weight HI) (Fig. 10; Supplementary Fig. S12).

## Discussion

Because FACE experiments are a great platform to study sink–source relations in crops under field conditions (e.g. Ainsworth *et al*., 2004; Ruiz-Vera *et al*., 2017; Bishop *et al*., 2018), we used this technology to evaluate the capacity of cassava storage roots to accumulate carbohydrates when photosynthesis is stimulated by elevated [CO_2_], which we hypothesized would prevent down-regulation of cassava photosynthesis. Our results supported our hypothesis because we found that the strong sink capacity of cassava roots enabled the storage of a large amount of carbohydrate that was associated with no down-regulation of photosynthesis and increased yield. In addition, iWUE increased due to the decrease in *g*_s_ associated with elevated [CO_2_]. Importantly from a nutritional perspective, N and protein content in leaves and HCN content in roots did not change at elevated [CO_2_]. Finally, our results provide insight into understanding cassava’s responses to an enriched CO_2_ environment, which may help guide the adaptation of cassava for future conditions.

### Elevated [CO_2_] increases photosynthesis with no signs of photosynthetic down-regulation after storage root initiation

Elevated [CO_2_] stimulated photosynthesis in the cultivars evaluated from 23% to 41% (Table 1; Supplementary Table S2). This range is similar to the range observed for cassava in cv. TMS60444 (15–53%; Rosenthal *et al*., 2012) and in other C_3_ crops grown at elevated [CO_2_] FACE conditions (e.g. Ainsworth and Long, 2005; Ainsworth and Rogers, 2007; Leakey *et al*., 2009; Bishop *et al*., 2014, 2018). C_3_ photosynthesis at ambient [CO_2_] is commonly Rubisco limited (i.e. *A* increases linearly with *C*_i_), whereas under elevated [CO_2_] (>570 μmol mol^−1^), C_3_ photosynthesis limitation usually occurs at the transition between the limitations imposed by Rubisco and ribulose 1,5-bisphosphate (RuBP) regeneration (inflection point of the *A*/*C*_i_ response curve) or at RuBP regeneration (where *A* no longer increases with increasing *C*_i_) (Ainsworth and Rogers, 2007; Rosenthal *et al*., 2012; Bishop *et al*., 2018; De Souza *et al*., 2019). On DOY 195, the 50% increase in *C*_i_ in elevated [CO_2_] gave the smallest increases in *A* when the rates of *A* were the highest for ambient [CO_2_]. Consequently, it is possible that *A* at ambient [CO_2_] was approaching limitation by RuBP regeneration. The highest increase in *A* due to elevated [CO_2_] was observed at the end of the growing season, which surprisingly coincided with the smallest increase in *C*_i_ (36%; Supplementary Fig. S1).

 Down-regulation of photosynthetic capacity can occur when carbohydrates accumulate in the leaves due to increased photosynthesis and/or inadequate sink capacity (Arp, 1991; Stitt, 1991; Long *et al*., 2004; Ainsworth *et al*., 2004; Rogers and Ainsworth, 2006; Burnett *et al*., 2016; Ruiz-Vera *et al*., 2017). This is typically observed *in vivo* as a decrease in apparent *V*_cmax_ and apparent *J*_max_ parameters (Long *et al*., 2004; Leakey *et al*., 2009). At the beginning of the growing season, we detected a small decrease in apparent *V*_cmax_ (–7%; DOY 199–202) in three of the eight cultivars (TME7, TMS98/0505, and TMS98/0581: Supplementary Fig. S2; Supplementary Table S2). At this stage, plants were 2 months old and storage roots were just initiating, with an average fresh weight of ~40 g (estimated from harvest of TME7 and TME419 roots on DOY 206). While *V*_cmax_ (i.e. when *g*_m_ was included in the calculation for *C*_c_) also decreased (10.5%), the difference was not statistically significant. Because elevated [CO_2_] did not affect *g*_m_ in these cultivars during this time of the season (DOY 199–202; Supplementary Table S2), the lack of statistical significance could reflect the higher variation observed in the values of *V*_cmax_ (standard error four times higher than in apparent *V*_cmax_). The reduced apparent *V*_cmax_ observed here in the beginning of the season agrees with previous studies showing similar responses for African cultivars in early stages (i.e. <2-month-old plants) of cassava development (De Souza and Long, 2018; De Souza *et al*., 2019). This also agrees with a previous FACE experiment showing transient photosynthetic acclimation in the beginning of the season for cv. TMS60444 (Rosenthal *et al*., 2012). During the following phases of cassava development, we observed similar *V*_cmax_, *J*_max_, apparent *V*_cmax_ and apparent *J*_max_ values across the cultivars, suggesting that once the storage roots start to accumulate biomass (i.e. ~3 months after planting; De Souza *et al*., 2017), cassava plants can fully utilize the carbohydrates produced by photosynthesis at elevated [CO_2_]. Supporting this interpretation, leaf starch accumulation under elevated [CO_2_] at the beginning of the season was more than double the amount later in the season even as the starch turnover rates were ~50% slower (Table 1; Fig. 8; Supplementary Fig. S10). As the season progressed, both TSC and starch turnover rates increased at elevated [CO_2_] and, by the end of the growing season, starch turnover was >150% the rates for ambient [CO_2_] plants (Fig. 8). This is also consistent with increased storage root biomass observed in plants under elevated [CO_2_] (Fig. 10). Irrespective of the [CO_2_] and time of measurement, starch content in leaves was more than double at the beginning of the season compared with the end of the season (Supplementary Fig. S10), and still photosynthetic down-regulation was not observed. This indicates that the maximum capacity of leaves to store carbohydrates was not reached during this experiment, helping to explain how cassava maintained high photosynthetic capacity at both ambient and elevated [CO_2_]. Data for TSC were not collected from DOY 195–196; however, increases in leaf TSC seem to associate with increases in leaf starch content, according to previous studies conducted in soybean at elevated [CO_2_] under FACE conditions (Rogers and Ainsworth, 2006; Ainsworth *et al*., 2007). Consequently, it is possible that TSC was also higher at the beginning of the season compared with later in the season.

### Elevated [CO_2_] increases iWUE in cassava over the entire season

Under elevated [CO_2_], *g*_s_ is nearly always lower, leading to improved iWUE (Leakey *et al*., 2009; Ainsworth and Long, 2005; Ainsworth and Rogers, 2007). In this study, the highest *g*_s_ values were observed at the beginning of the season (DOY 195), which corresponded to the highest reductions in *g*_s_ (–24%) under elevated [CO_2_]. The largest increase in iWUE (+70%) occurred later in the season (DOY 269) and did not correspond to the largest decrease in *g*_s_. Because *g*_s_ at ambient [CO_2_] on DOY 269 was lower than *g*_s_ at elevated [CO_2_] on DOY 195 (Supplementary Fig. S1), the already low *g*_s_ values (0.2 mol m^−2^ s^−1^ at ambient [CO_2_]) might have also influenced the magnitude of the reduction in *g*_s_ at elevated [CO_2_]. During the middle of the season, elevated [CO_2_] did not affect *g*_s_ in any of the cultivars. Nevertheless, iWUE significantly increased (48%) in this period due to the stimulation of *A* under elevated [CO_2_] (Supplementary Fig. S1; Supplementary Table S2). The lack of *g*_s_ reduction during this time, and probably the lack of change in *g*_m_ obtained from the *A/C*_i_ data, could have contributed to the large increases in *C*_i_ at elevated [CO_2_], which were >60% for three cultivars (Supplementary Fig. S1). Interestingly, cultivars with the largest increases in *A* at elevated [CO_2_] were not those that had the largest increases in *C*_i_. The variation of *g*_s_ across the cultivars was 44% at ambient and 63% at elevated [CO_2_], which was higher than the variation in *A* of 21% at ambient and 27% at elevated [CO_2_] (Supplementary Fig. S1). Despite variation in *A* and *g*_s_ across cultivars, iWUE increased at elevated [CO_2_] in all cultivars throughout the season, ranging from 44% to 68%, depending on the cultivar (Fig. 3). With climate change, more frequent droughts are expected in the sub-Sahara African region (Rosenthal *et al*., 2012; Serdeczny *et al*., 2017), and under these conditions cassava may benefit from increased iWUE to increase productivity. However, changes in iWUE do not always translate to similar changes in whole-plant WUE (Medrano *et al*., 2015). For example, increases in iWUE under elevated [CO_2_] occur together with slightly higher canopy temperature and greater biomass production, both of which can increase transpiration (Bernacchi *et al*., 2007). Consequently, whether the overall 58% increase in iWUE found in cassava at elevated [CO_2_] will result in WUE improvements at the whole-plant and crop level remains to be evaluated.

### Elevated [CO_2_] did not affect leaf N or HCN content in cassava plants

Rubisco is the most abundant protein in the world (Ellis, 1979; Bar-On and Milo, 2019), and the amount of N invested in Rubisco can be as much as 25% of total protein in C_3_ plants (Sage *et al*., 1987; Spreitzer and Salvucci, 2002; Halpern *et al*., 2019). When C_3_ plants are grown at elevated [CO_2_], leaf N and protein content often decline in non-leguminous species, and Rubisco content may decline by up to 20% on a leaf area basis (Drake *et al*., 1997; Gleadow *et al*., 1998; Ainsworth *et al*., 2002; Long *et al*., 2004; Taub *et al*., 2008), which leads to down-regulation of photosynthesis. Additionally, C_3_ plants grown at elevated [CO_2_] most often have a higher photosynthetic N use efficiency due to a higher amount of C fixed per unit of N in the leaf (Leakey *et al*., 2009).

Consistent with our observation that photosynthesis was not down-regulated in cassava, leaf N and protein content did not change, despite an ~8% increase in the C:N ratio under elevated [CO_2_] (Fig. 7; Table 1; Supplementary Figs S7, S8; Supplementary Table S3). In the previous cassava FACE experiment, leaf N decreased at elevated [CO_2_] (Rosenthal *et al*., 2012). Adequate N supply to support strong sink development can ameliorate the down-regulation of photosynthesis under elevated [CO_2_] (Gleadow *et al*., 1998; Ruiz-Vera *et al*., 2017; Halpern *et al*., 2019). However, N fertilization in cassava is not common practice in sub-Saharan Africa due to high fertilization costs (Druilhe and Barreiro-Hurlé, 2012). The amount of N fertilization applied in this experiment (i.e. 84 kg N ha^−1^) is within the range to maximize cassava yield at current [CO_2_] (Howeler, 2002, 2014; Biratu *et al*., 2018). Under a scenario of low N fertilization and elevated [CO_2_], sink development may be constrained and a down-regulation of photosynthesis might be observed, but this still needs to be tested.

Few studies have analyzed the changes in the toxicity of cassava under elevated [CO_2_]. Moreover, results have been contradictory, with increases (Gleadow *et al*., 2009*a*), decreases (Forbes *et al*., 2020), or no change (Rosenthal *et al*., 2012) in the amount of cyanide-containing compounds in the tissues of plants grown at elevated [CO_2_]. These differing results could be due to differences in the experimental conditions or cultivar-specific responses. In this study, elevated [CO_2_] decreased HCN of the leaves in two cultivars at certain times during the season but did not affect HCN in the peel and core of the storage roots in any of the eight cultivars (Table 1; Fig. 9; Supplementary Fig. S11; Supplementary Table S3). Thus, the nine cultivars grown under FACE conditions suggest that HCN content will remain the same or decrease. Nevertheless, it will be important to investigate possible changes in plants older than 4 months and under low or no N fertilization conditions to fully understand how cassava toxicity will vary with elevated [CO_2_] in mature plants and under practices followed in sub-Saharan Africa.

### CO_2_ stimulation of growth, above- and below-ground biomass, and resource allocation varies among the cassava cultivars

LAI values obtained by the end of the experiment in both [CO_2_] treatments were higher (Fig. 6; Supplementary Fig. S6) than those obtained in the previous cassava FACE experiment with values of 3 for plants grown at ambient [CO_2_] and 3.5 for plants grown at elevated [CO_2_] (Rosenthal *et al*., 2012). The LAI values collected in this experiment were also higher than what is considered the optimum LAI for root bulking rates in cassava, according to Cock *et al*. (1979). In both of these earlier experiments, plants were located every 1 m, while this experiment used 0.7 m spacing. Plant spacing can in part explain the high values of LAI. LAI is higher when plant spacing decreases or plant density increases for cassava grown at adequate soil N conditions and can exceed 7.5 at plant spacing similar to that used in this study (Streck *et al*., 2014). Additionally, high LAI may partially explain why the percentage increase in above- and below-ground biomass was lower than in the other FACE experiment (Rosenthal *et al*., 2012), for example due to low light penetration deeper into the canopy.

Overall, above- and below-ground biomass increased at elevated [CO_2_], which corresponded to higher LAI, height, number of leaves, and number of branches (Figs 6, 10; Table 1; Supplementary Figs S6, S12). The range of 22–39% stimulation in storage root fresh weight under elevated [CO_2_] is similar to the increase observed in potato (+40%; Miglietta *et al*., 1998), but lower than values obtained for a different cassava cultivar at the same experimental site (~90%; Rosenthal *et al*., 2012), perhaps due to a higher early season bulking rate in this cultivar. The fact that roots maintained higher biomass after drying at elevated compared with ambient [CO_2_] indicates a high capacity of the roots to accumulate carbohydrate. Because dry roots achieved a higher percentage increase in biomass (from 25% to 51% in four cultivars; Supplementary Fig. S12) than fresh roots, some cassava cultivars had lower water content in the roots when grown at elevated [CO_2_] than at ambient [CO_2_] (TMS98/0505 and TMS98/0581; Fig.10; Supplementary Fig. S12). With the exception of TMS98/002, the cultivars with the highest stimulation in dry storage roots were not the same as the cultivars with the highest increases in photosynthesis (Fig. 3; Supplementary Fig. S12). This may reflect cultivar-specific efficiency to translocate carbohydrates to the storage roots and/or differences in canopy structure that alter the relationship between leaf *A* and canopy *A*. For example, elevated [CO_2_] delayed branch development in TME7, indicating priority in investing resources in roots over branches. Despite a low stimulation of leaf *A* under elevated [CO_2_], TMS98/0581 exhibited the largest elevated [CO_2_] increase in storage root fresh and dry weight (+39% and +51%, respectively; Fig. 10; Supplementary Fig. S12). This cultivar was also the only one to have high LAI and AGB but similar leaf number at elevated [CO_2_]. Despite more efficient partitioning to storage root biomass under elevated [CO_2_] than the other cultivars, elevated [CO_2_] might have also altered TMS98/0581 canopy architecture that allowed higher light interception efficiency (e.g. larger leaves).

Despite a similar fresh weight of AGB at ambient [CO_2_], TMS98/0002 and TMS30572 had contrasting allocation patterns to roots at elevated [CO_2_]. At elevated [CO_2_], TMS98/0002 increased storage root fresh and dry weight by 33% but AGB fresh weight by only 8% (Fig. 10; Supplementary Fig. S12). In contrast, TMS30572 showed a small and non-significant increase in storage root fresh and dry weight at elevated [CO_2_] (+11% and +5%, respectively), while its fresh and dry weight of AGB significantly increased by 25% and 37%, respectively (Fig. 10; Supplementary Fig. S12). These differences in CO_2_ response and partitioning reflect the intraspecific variations that are particular to the genetic background of each cultivar and suggest they could be explored further to gain a better understanding of how biomass allocation might be improved for future environmental conditions. Interestingly, the effects of elevated [CO_2_] on biomass partitioning of cassava did not alter HI, which was the same for plants grown at ambient and elevated [CO_2_] (Fig. 10; Supplementary Fig. S12). Similar results have been observed in an open-top chamber potato study (Donnelly *et al*., 2001) but differed from another potato study conducted under FACE conditions (Miglietta *et al*., 1998).

In terms of food security, the high sink and photosynthetic capacity of cassava allowed a promising stimulation of yield and overall biomass under elevated [CO_2_]. Whether this stimulation will increase or be maintained through a complete growing season (~10 months) and with less or no N addition in African soils still needs to be evaluated. In this study, cassava plants grew in an organically rich soil (Flanagan/Drummer soil) that was fertilized with N and without water or temperature stress (maximum temperature range: 21–35 °C between DOY 154 and 274 with the exception of one day; Fig. 1). Consequently, this study provides the first results of how African cassava cultivars will respond to elevated [CO_2_] under what may be nearly optimal growing conditions.

### Conclusion

As the demand for cassava storage roots increases, productivity gains are needed. To know if enhancing cassava’s photosynthetic efficiency will have the potential to increase yields, it is important to know if cassava storage roots have the capacity to use additional carbohydrate. We evaluated the sink strength of cassava when grown under elevated [CO_2_] conditions, which increased photosynthetic efficiency, and found high sink capacity in cassava roots coupled with high photosynthetic rates resulting in greater root biomass under elevated [CO_2_]. These findings support the notion that cassava yields can be increased by improving photosynthetic efficiency. Above- and below-ground biomass allocations varied among cultivars, increasing root biomass more in certain cultivars. These differences in biomass partitioning can facilitate the identification of promising cultivars for breeding to increase cassava yield. Cassava grown at elevated [CO_2_] also exhibited improved water and N use efficiency, which are highly desirable traits in African agriculture where drought conditions are expected to be more common and N fertilization is uncommon. Under elevated [CO_2_], cassava leaf protein content and toxicity in leaves and roots were unchanged. These findings suggest that breeding for high photosynthetic efficiency in cassava might be possible without changes in other important characteristics for its consumption.

## Supplementary data

The following supplementary data are available at *JXB* online.

Dataset S1. Raw data to which the *A*/*C*_i_ curves at 28 °C were fit.

Figs S1 and S5–S11. Per day average of gas exchange and growth parameters, SLA, leaf N, C:N, leaf protein and carbohydrates content, and HCN from the eight cultivars of cassava at ambient and elevated [CO_2_].

Fig. S2. Fitted responses of *A*/*C*_i_ curves at 28 °C from the eight cultivars of cassava grown at ambient and elevated [CO_2_] for the measurements conducted on DOY 199–202.

Fig. S3. Fitted responses of *A*/*C*_i_ curves at 28 °C from the eight cultivars of cassava grown at ambient and elevated [CO_2_] for the measurements conducted on DOY 226–229. Fig. S4. Fitted responses of *A*/*C*_i_ curves at 28 °C from the eight cultivars of cassava grown at ambient and elevated [CO_2_] for the measurements conducted on DOY 267–269.

Fig. S12. Dry weight biomass of eight cultivars of cassava grown at ambient and elevated [CO_2_] during the final harvest.

 Fig. S13. Tuber yield of eight cultivars of cassava grown at ambient and elevated [CO_2_] during the final harvest.

Table S1. Principal component loadings from PC1 and PC2 for each of the parameters.

Tables S2 and S3. Statistical analysis of the daily average for each parameter.

eraa459_suppl_Supplementary_File001

eraa459_suppl_Supplementary_File002

## Data Availability

The data supporting the findings of this study are available from the corresponding author (DRO) upon request.

## Abbreviations

A: photosynthetic carbon uptake
*C*_i_ [CO_2_]: inside the leaf
[CO_2_]: carbon dioxide concentration
DOY: day of the year
FACE: free-air CO_2_ enrichment
*g*_m_: mesophyll conductance
*g*_s_: stomatal conductance
HCN: hydrogen cyanide
HI: harvest index
iWUE: intrinsic water use efficiency
*J*_max_: maximum rate of photosynthetic electron transport
LAI: leaf area index
SLA: specific leaf area
TSC: total soluble carbohydrate
*V*_cmax_: maximum rate of carboxylation by Rubisco
