# Supplementary material for: High sink strength prevents photosynthetic down-regulation in cassava grown at elevated CO2 concentration
Source: J Exp Bot. 2020 Oct 12;72(2):542–60. doi: 10.1093/jxb/eraa459 (PMC7853607; doi:10.1093/jxb/eraa459)
Supplement: eraa459_suppl_Supplementary_File002 [file eraa459_suppl_supplementary_file002.pdf]

SUPPLEMENTARY INFORMATION

HIGH SINK-STRENGTH PREVENTS PHOTOSYNTHETIC DOWN-REGULATION IN CASSAVA GROWN AT ELEVATED CO_2 CONCENTRATION

Ursula M. Ruiz-Vera[1], Amanda P. De Souza[1], Michael R. Ament[1], Roslyn M. Gleadow[2], Donald R. Ort[1,3*]

[1] Carl R. Woese Institute for Genomic Biology, University of Illinois at Urbana-Champaign, Urbana, Illinois, USA.

[2] School of Biological Sciences, Monash University, Clayton, Victoria, Australia

[3] Departments of Plant Biology and Crop Sciences, University of Illinois at Urbana-Champaign, Urbana, Illinois, USA.

*Corresponding author: Donald R. Ort: d-ort@illinois.edu

Dataset S1

Raw data to which the A/C_i curves at 28°C were fit from eight cultivars of cassava grown at ambient and elevated [CO_2]. See excel file.

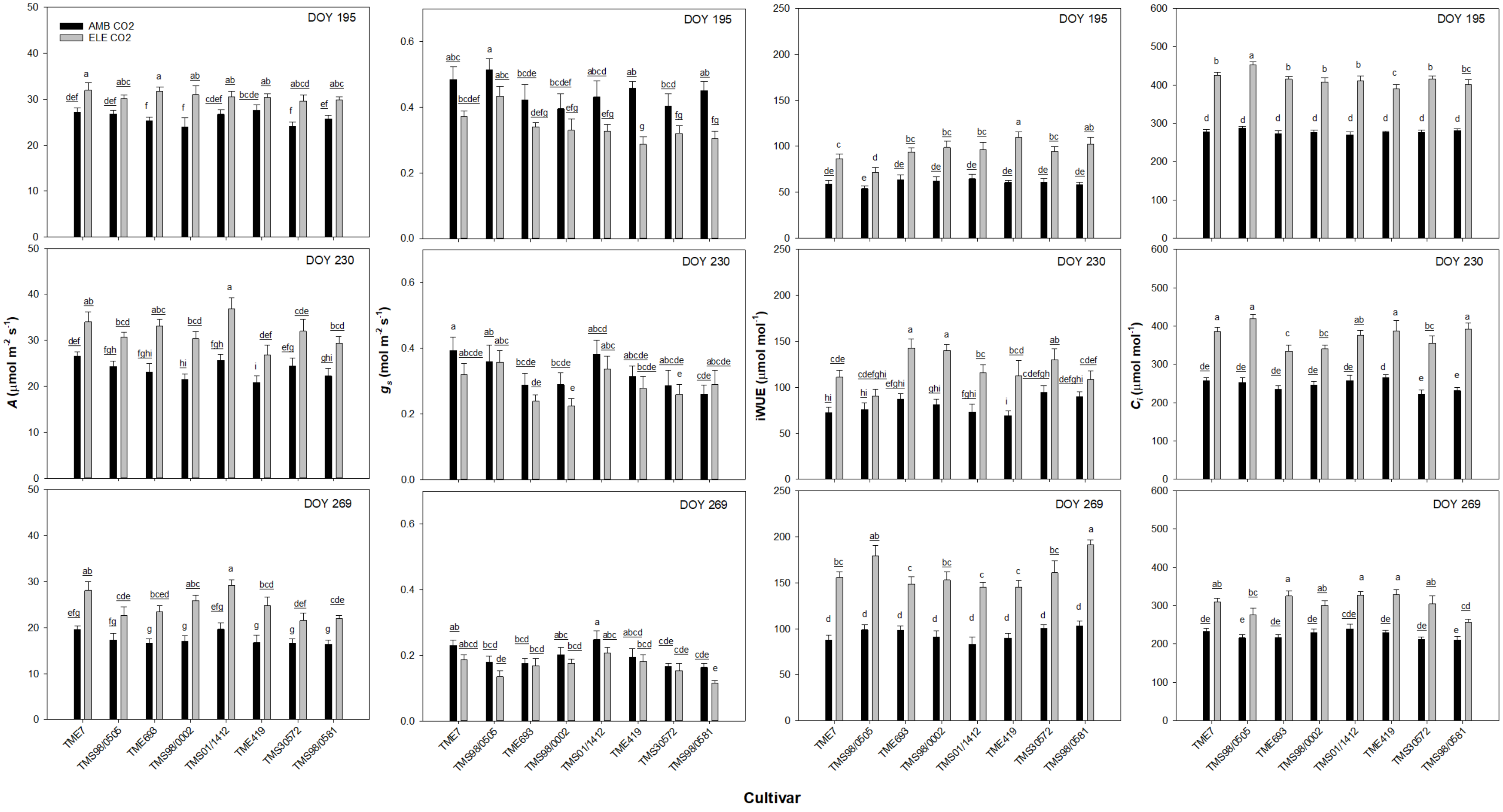

AMB CO2
ELE CO2
DOY 195
DOY 230
DOY 269
A (μmol m-2 s-1)
gs (mol m-2 s-1)
iWUE (μmol mol-1)
Ci (μmol mol-1)
TME7
TMS98/0505
TME693
TMS98/0002
TMS01/1412
TME419
TMS30572
TMS98/0581
Cultivar

Figure S1. Per day average of gas exchange parameters from eight cultivars of cassava at ambient (AMB CO2) and elevated [CO_2] (ELE CO2). Photosynthetic carbon uptake (*A*, μmol CO_2 m^{-2} s^{-1}), stomatal conductance (g_s, mol H_2O m^{-2} s^{-1}), intrinsic water use efficiency (iWUE, μmol mol^{-1}), and [CO_2] inside the leaf (C_i, μmol mol^{-1}) from eight cultivars of cassava at grown ambient (AMB CO2) and elevated [CO_2] (ELE CO2). The days of the year (DOY) when the measurements were taken are indicated in each panel. Error bars are mean ± standard error (SE; n=4). Treatments with different letters represent significant differences ($p<0.1$), underlying is used to help differentiate group of letters.

.

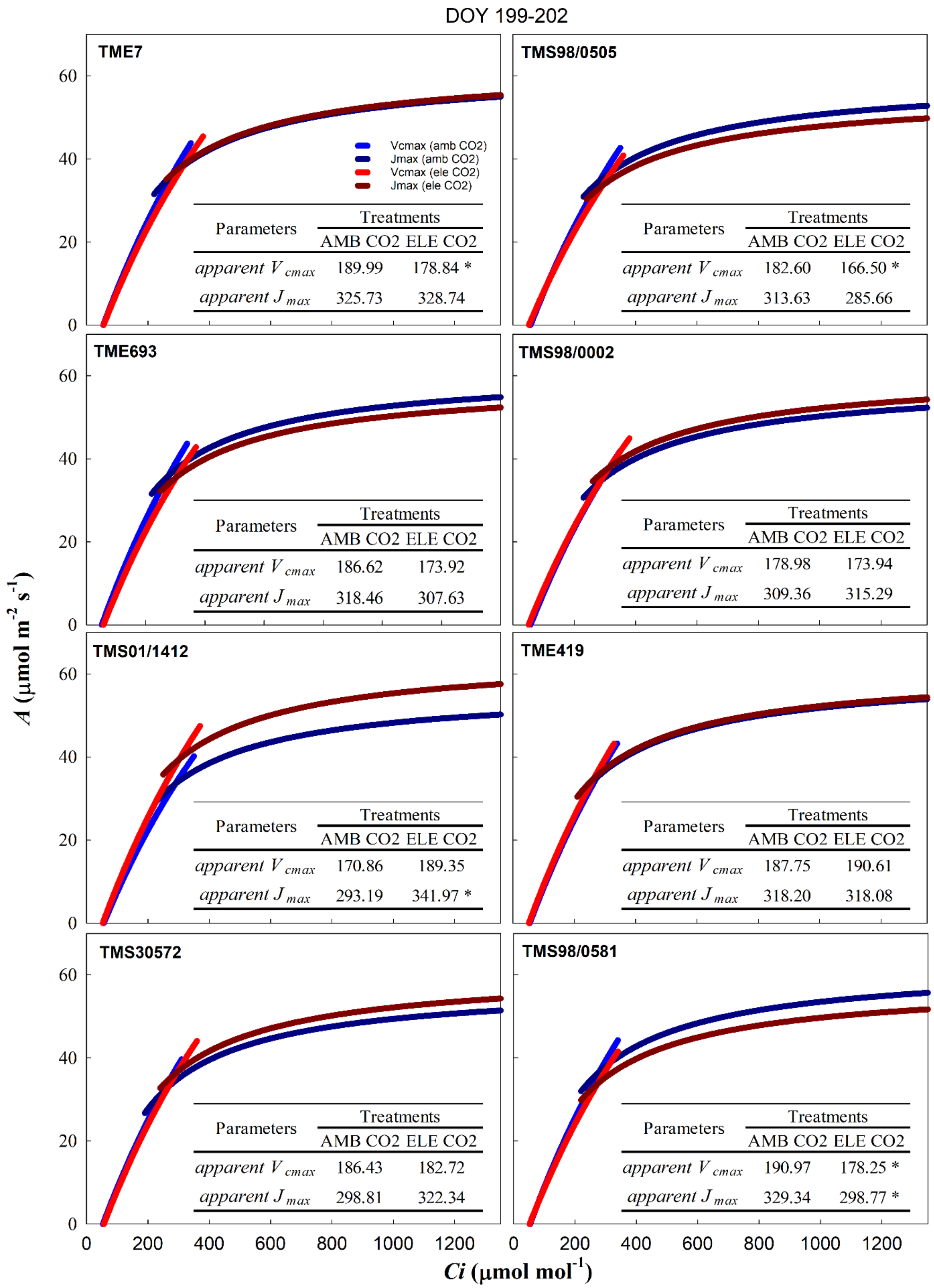
DOY 199-202
TME7
Vcmax (amb CO2)
Jmax (amb CO2)
Vcmax (ele CO2)
Jmax (ele CO2)
Parameters | Treatments: AMB CO2 | ELE CO2
apparent V cmax | 189.99 | 178.84 *
apparent J max | 325.73 | 328.74
TMS98/0505
Parameters | Treatments: AMB CO2 | ELE CO2
apparent V cmax | 182.60 | 166.50 *
apparent J max | 313.63 | 285.66
TME693
Parameters | Treatments: AMB CO2 | ELE CO2
apparent V cmax | 186.62 | 173.92
apparent J max | 318.46 | 307.63
TMS98/0002
Parameters | Treatments: AMB CO2 | ELE CO2
apparent V cmax | 178.98 | 173.94
apparent J max | 309.36 | 315.29
TMS01/1412
Parameters | Treatments: AMB CO2 | ELE CO2
apparent V cmax | 170.86 | 189.35
apparent J max | 293.19 | 341.97 *
TME419
Parameters | Treatments: AMB CO2 | ELE CO2
apparent V cmax | 187.75 | 190.61
apparent J max | 318.20 | 318.08
TMS30572
Parameters | Treatments: AMB CO2 | ELE CO2
apparent V cmax | 186.43 | 182.72
apparent J max | 298.81 | 322.34
TMS98/0581
Parameters | Treatments: AMB CO2 | ELE CO2
apparent V cmax | 190.97 | 178.25 *
apparent J max | 329.34 | 298.77 *
A (μmol m-2 s-1)
Ci (μmol mol-1)
0
20
40
60
0
200
400
600
800
1000
1200

Figure S2. Fitted responses of A/C_i curves at 28°C from eight cultivars of cassava grown at ambient (AMB CO2) and elevated [CO_2] (ELE CO2) during the day of the year (DOY) 199-202. The mean apparent V_{cmax} (μmol m^{-2} s^{-1}, SE <3.6-9.1>) and apparent J_{max} (μmol m^{-2} s^{-1}, se <8.1-17.8>) are indicated in the inserted tables. Significant differences are indicated with '*' (p<0.1). Ambient [CO_2] treatments are represented by blue lines and the elevated [CO_2] treatments by red lines. The raw data to which the lines are fit are in Supplementary Dataset S1. Mean leaf temperature for the raw data was between 30.7°C for TME7 and 31.8°C for TMS98/0581.

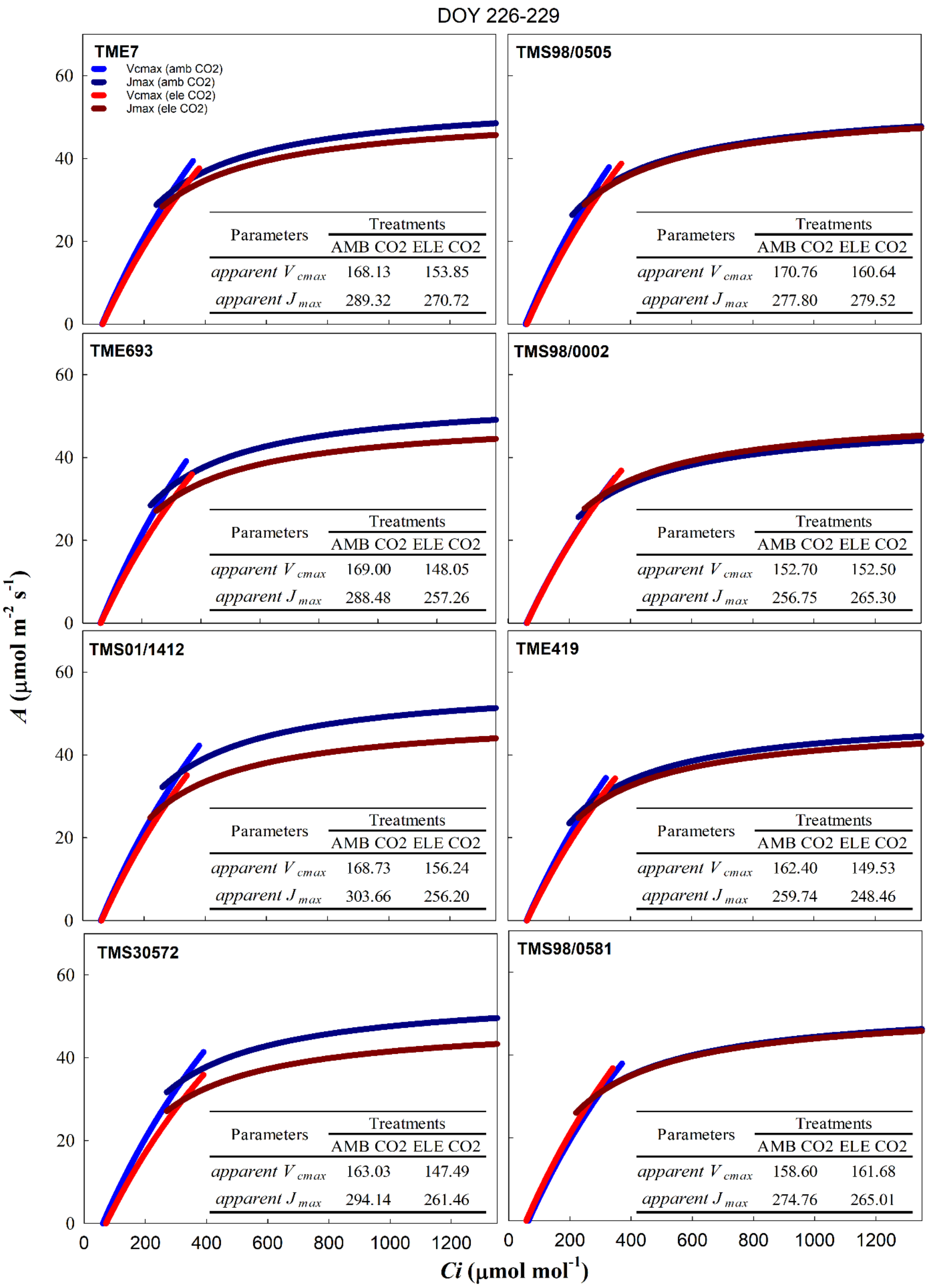
DOY 226-229
TME7
Vcmax (amb CO2)
Jmax (amb CO2)
Vcmax (ele CO2)
Jmax (ele CO2)
Parameters | Treatments | AMB CO2 | ELE CO2
apparent V cmax 168.13 153.85
apparent J max 289.32 270.72
TMS98/0505
apparent V cmax 170.76 160.64
apparent J max 277.80 279.52
TME693
apparent V cmax 169.00 148.05
apparent J max 288.48 257.26
TMS98/0002
apparent V cmax 152.70 152.50
apparent J max 256.75 265.30
TMS01/1412
apparent V cmax 168.73 156.24
apparent J max 303.66 256.20
TME419
apparent V cmax 162.40 149.53
apparent J max 259.74 248.46
TMS30572
apparent V cmax 163.03 147.49
apparent J max 294.14 261.46
TMS98/0581
apparent V cmax 158.60 161.68
apparent J max 274.76 265.01
A (μmol m-2 s-1)
Ci (μmol mol-1)
0
20
40
60
0
200
400
600
800
1000
1200

Figure S3. Fitted responses of A/C_i curves at 28°C from eight cultivars of cassava grown at ambient (AMB CO2) and elevated [CO_2] (ELE CO2) during the day of the year (DOY) 226-229. The mean apparent V_{cmax} (µmol m^{-2} s^{-1}, SE <5.6-16.2>) and apparent J_{max} (µmol m^{-2} s^{-1}, se <9.4-27.6>) are indicated in the inserted tables. Elevated CO2 had non-significant effect in apparent V_{cmax} and apparent J_{max}. Ambient [CO_2] treatments are represented by blue lines and the elevated [CO_2] treatments by red lines. The data to which the lines are fit are in Supplementary Dataset 1. Mean leaf temperature for the raw data was between 29.1°C for TMS30572 and 30.3°C for TME419.

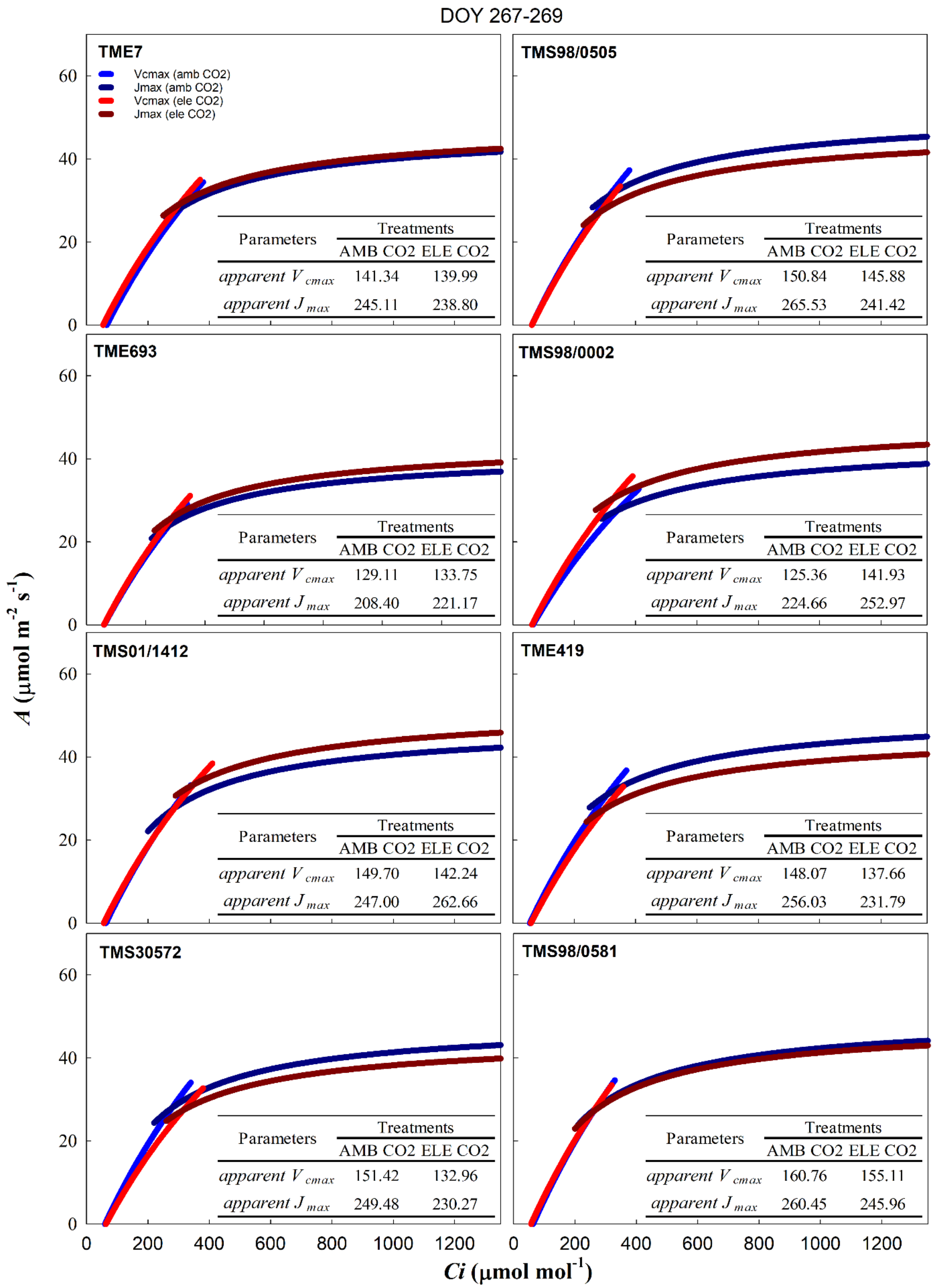
DOY 267-269
TME7
Vcmax (amb CO2)
Jmax (amb CO2)
Vcmax (ele CO2)
Jmax (ele CO2)
Parameters | Treatments: AMB CO2, ELE CO2
apparent V cmax 141.34 139.99
apparent J max 245.11 238.80
TMS98/0505
apparent V cmax 150.84 145.88
apparent J max 265.53 241.42
TME693
apparent V cmax 129.11 133.75
apparent J max 208.40 221.17
TMS98/0002
apparent V cmax 125.36 141.93
apparent J max 224.66 252.97
TMS01/1412
apparent V cmax 149.70 142.24
apparent J max 247.00 262.66
TME419
apparent V cmax 148.07 137.66
apparent J max 256.03 231.79
TMS30572
apparent V cmax 151.42 132.96
apparent J max 249.48 230.27
TMS98/0581
apparent V cmax 160.76 155.11
apparent J max 260.45 245.96
A (μmol m-2 s-1)
Ci (μmol mol-1)
0
20
40
60
200
400
600
800
1000
1200

Figure S4. Fitted responses of A/C_i curves at 28°C from eight cultivars of cassava grown at ambient (AMB CO2) and elevated [CO_2] (ELE CO2) during the day of the year (DOY) 267-269. The mean apparent V_{cmax} (µmol m^{-2} s^{-1}, SE <3.2-12.9>) and apparent J_{max} (µmol m^{-2} s^{-1}, se <6.7-19.5>) are indicated in the inserted tables. Elevated CO2 had non-significant effect in apparent V_{cmax} and apparent J_{max}. Ambient [CO_2] treatments are represented by blue lines and the elevated [CO_2] treatments by red lines. The data to which the lines are fit are in Supplementary Dataset 1. Mean leaf temperature for the raw data was between 30.7°C for TME7 and 32.5°C for TMS98/0581

.

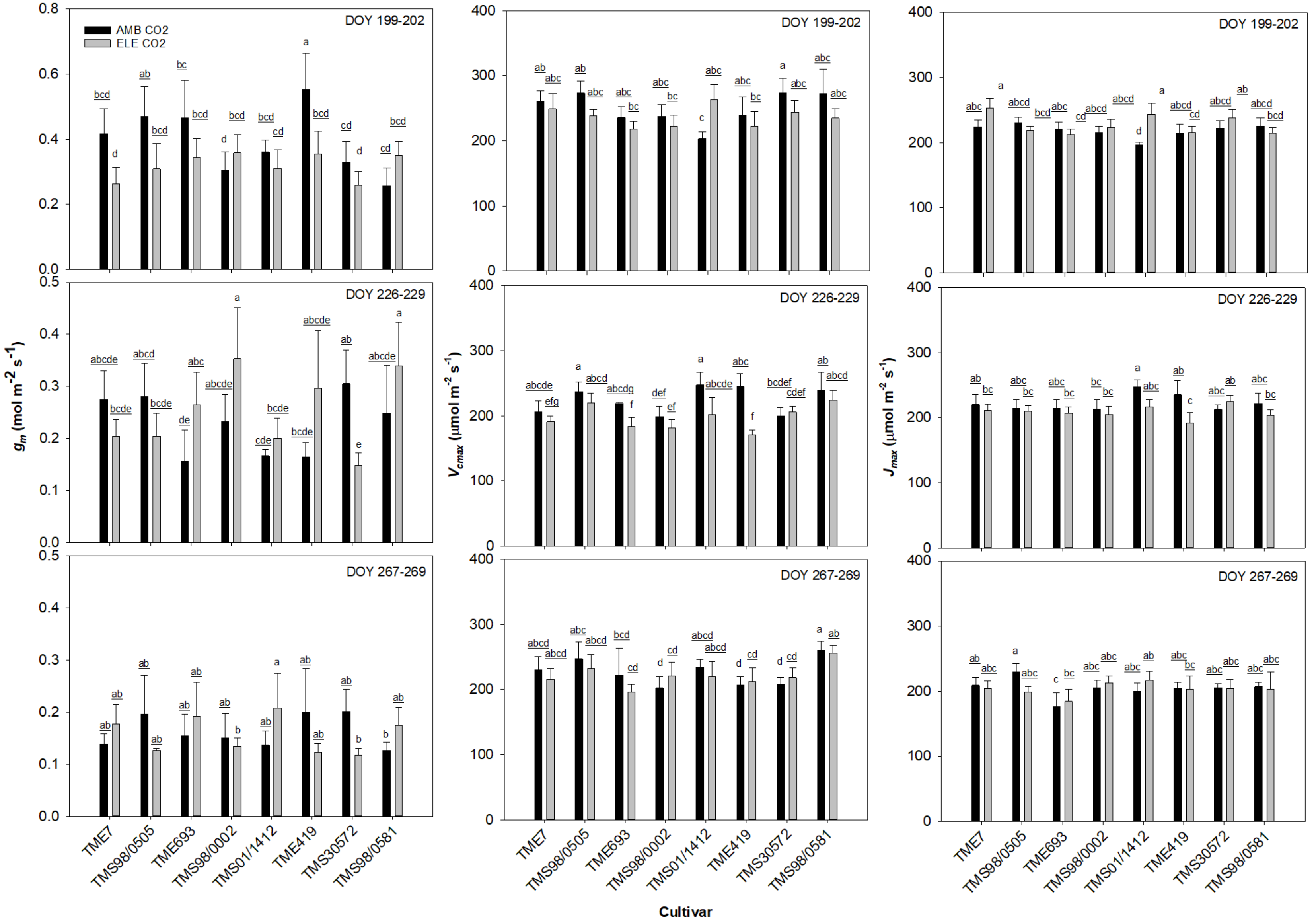
AMB CO2
ELE CO2
DOY 199-202
DOY 226-229
DOY 267-269
g_m (mol m^{-2} s^{-1})
V_{cmax} (µmol m^{-2} s^{-1})
J_{max} (µmol m^{-2} s^{-1})
TME7
TMS98/0505
TME693
TMS98/0002
TMS01/1412
TME419
TMS30572
TMS98/0581
Cultivar

Figure S5. Per day average of photosynthetic parameters. Mesophyll conductance (g_m, mol m^2 s^{-1}), the maximum carboxylation rate by Rubisco (V_{cmax}, µmol m^{-2} s^{-1}) and the regeneration of ribulose-1,5-biphosphate controlled by the electron transport rate (J_{max}, µmol m^{-2} s^{-1}) at 28°C from eight cultivars of cassava grown at ambient (AMB CO2) and elevated [CO_2] (ELE CO2). The days of the year (DOY) when the measurements were taken are indicated in each panel. Error bars are mean ± standard error (SE; n=4). Treatments with different letters represent significant differences (p<0.1), underlying is used to help differentiate group of letters.

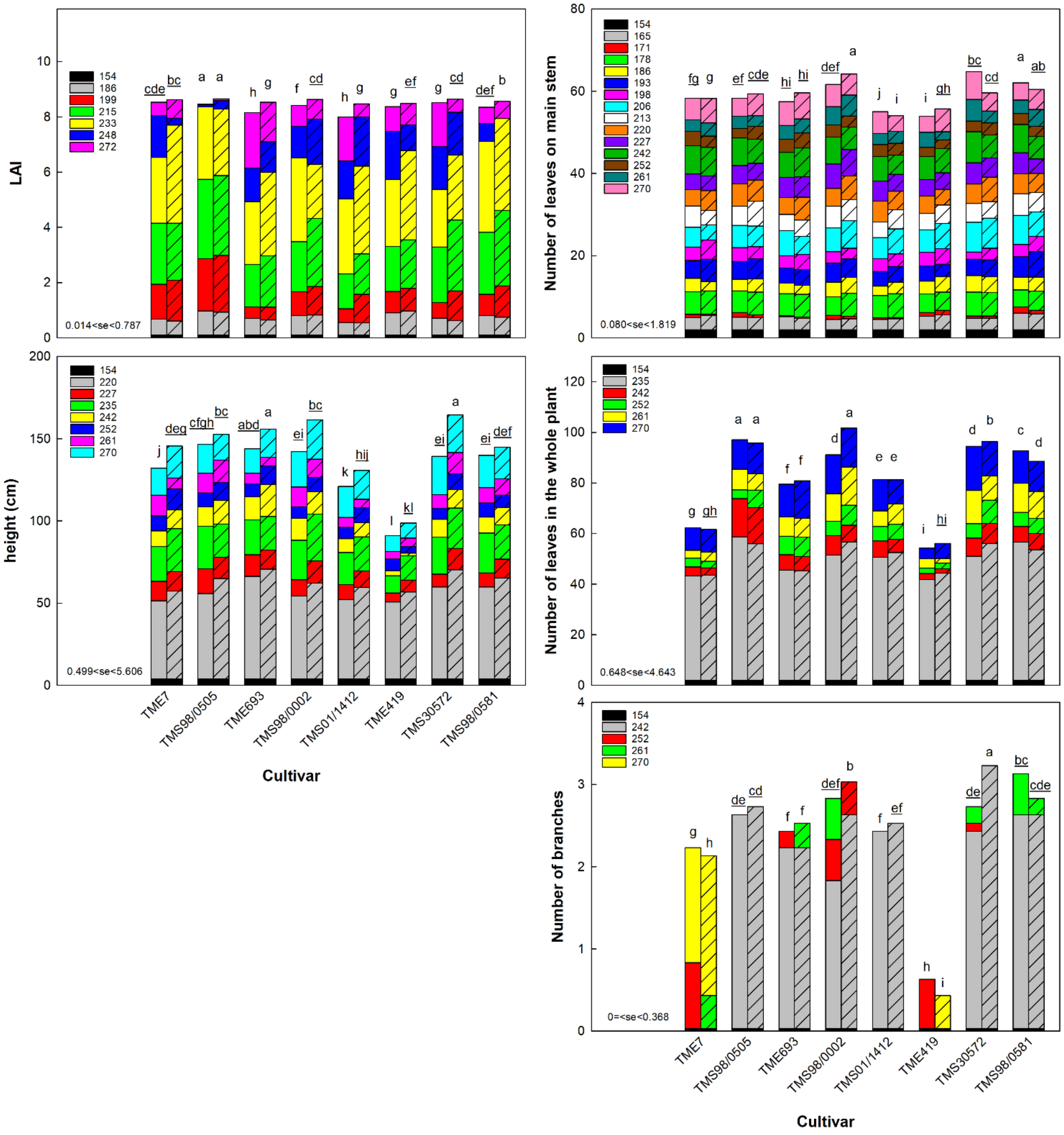

Figure S6. Per day average of growth parameters taken in eight cultivars of cassava grown at ambient (AMB CO2) and elevated [CO_2] (ELE CO2). Leaf area index (LAI; m^2 m^{-2}), height (cm), number of leaves in the main stem and whole plant, and number of branches. The days of the year (DOY) when the measurements were taken are indicated in each panel and are represented with a different color. DOY 154 is the starting day or day of planting. For LAI, only 6 of 11 measurements days are shown. Error bars are mean ± standard error (SE; n=4).

Treatments with different letters represent significant differences ($p<0.1$) for the seasonal results, underlying is used to help differentiate group of letters

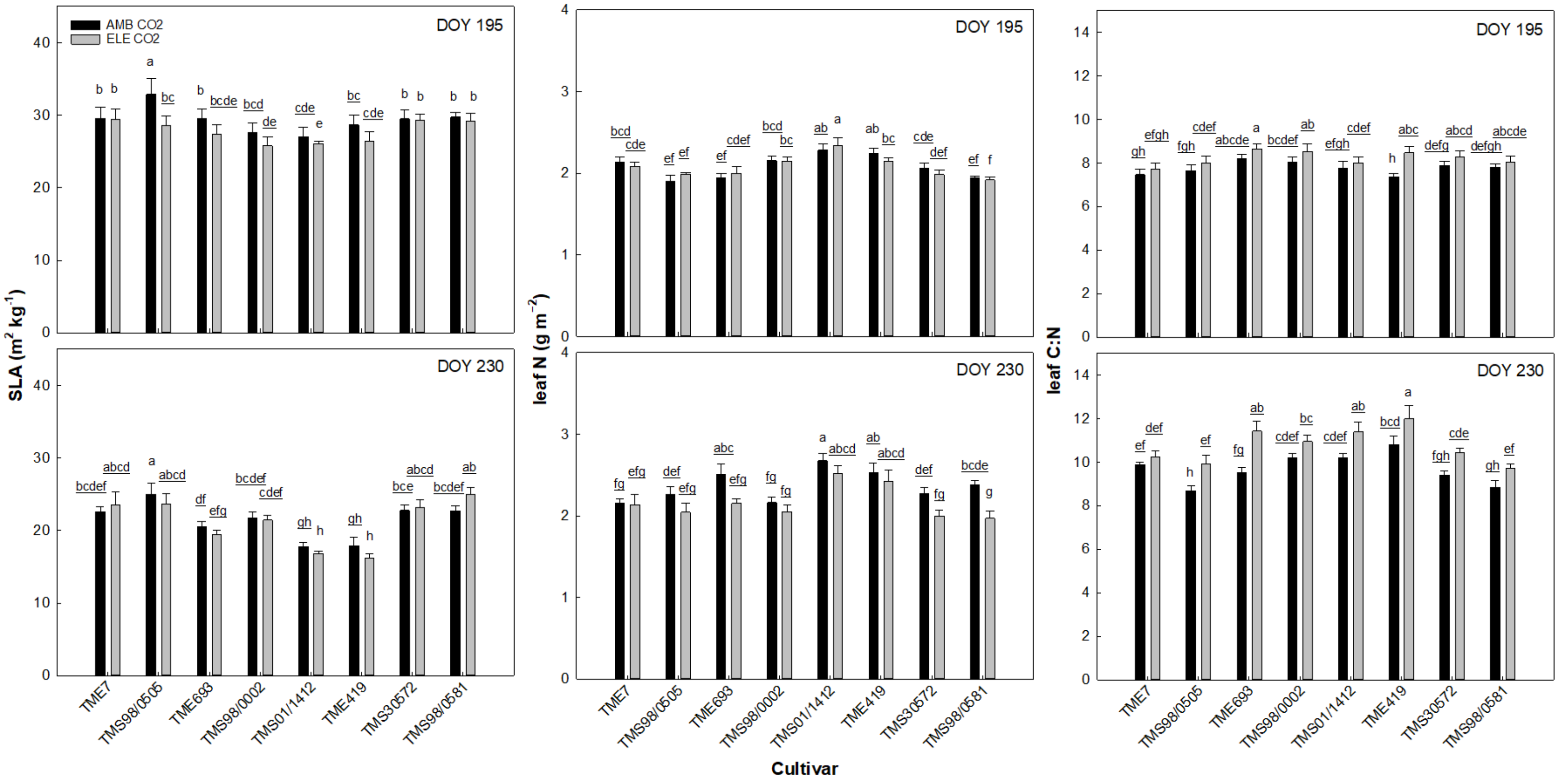

AMB CO2
ELE CO2
DOY 195
DOY 230
SLA (m^2 kg^{-1})
leaf N (g m^{-2})
leaf C:N
Cultivar
TME7
TMS98/0505
TME693
TMS98/0002
TMS01/1412
TME419
TMS30572
TMS98/0581

Figure S7. Per day average of the specific leave area (SLA; $m^2 kg^{-1}$), leaf nitrogen ($g m^{-2}$), and carbon vs. nitrogen ratio (C:N) from eight cultivars of cassava grown at ambient (AMB CO2) and elevated [CO_2] (ELE CO2). The days of the year (DOY) when the measurements were taken are indicated in each panel. Error bars are mean ± standard error (SE; n=4). Treatments with different letters represent significant differences ($p<0.1$), underlying is used to help differentiate group of letters.

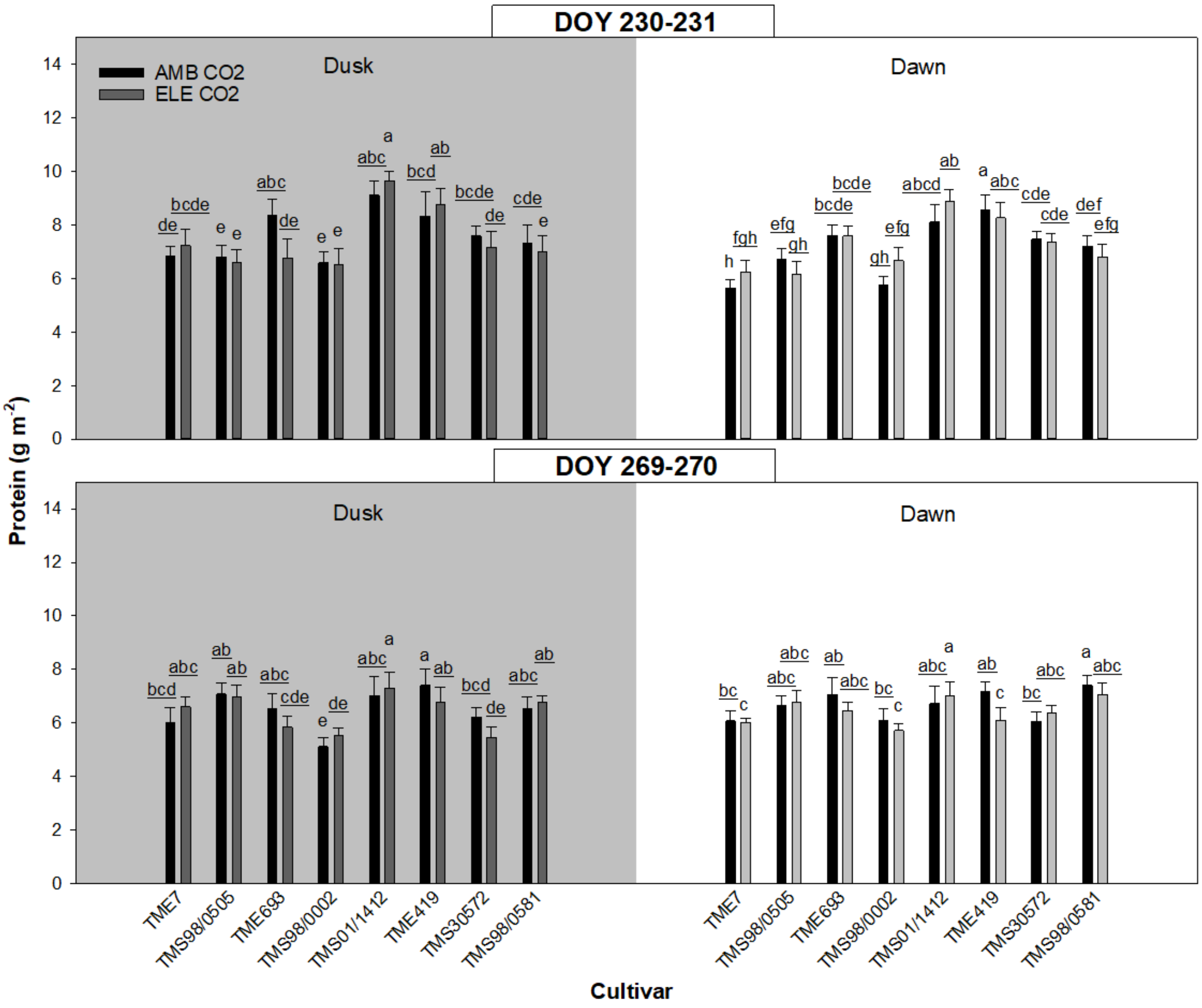

Figure S8. Per day average of the leaf protein content (g m^{-2}) at dusk and dawn from eight cultivars of cassava grown at ambient (AMB CO2) and elevated [CO_2] (ELE CO2). The days of the year (DOY) when the measurements were taken are indicated at the top each of panel. Error bars are mean ± standard error (SE; n=4). Treatments with different letters represent significant differences (p<0.1), underlying is used to help differentiate group of letters.

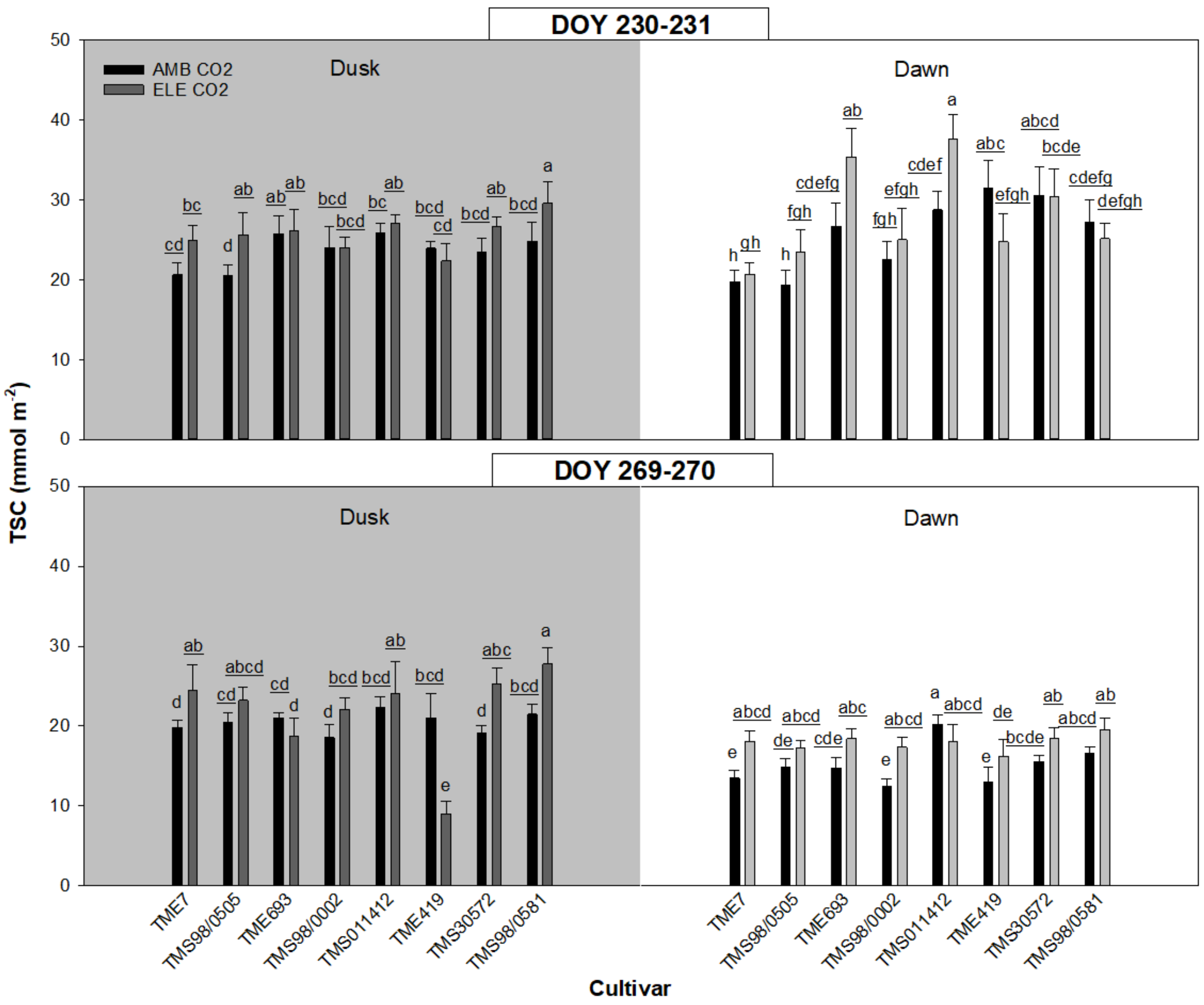

Figure S9. Per day average of the total soluble carbohydrates (TSC, mmol m^{-2}) at dusk and dawn from eight cultivars of cassava grown at ambient (AMB CO2) and elevated [CO_2] (ELE CO2). The days of the year (DOY) when the measurements were taken are indicated at the top each of panel. Error bars are mean ± standard error (SE; n=4). Treatments with different letters represent significant differences (p<0.1), underlying is used to help differentiate group of letters.

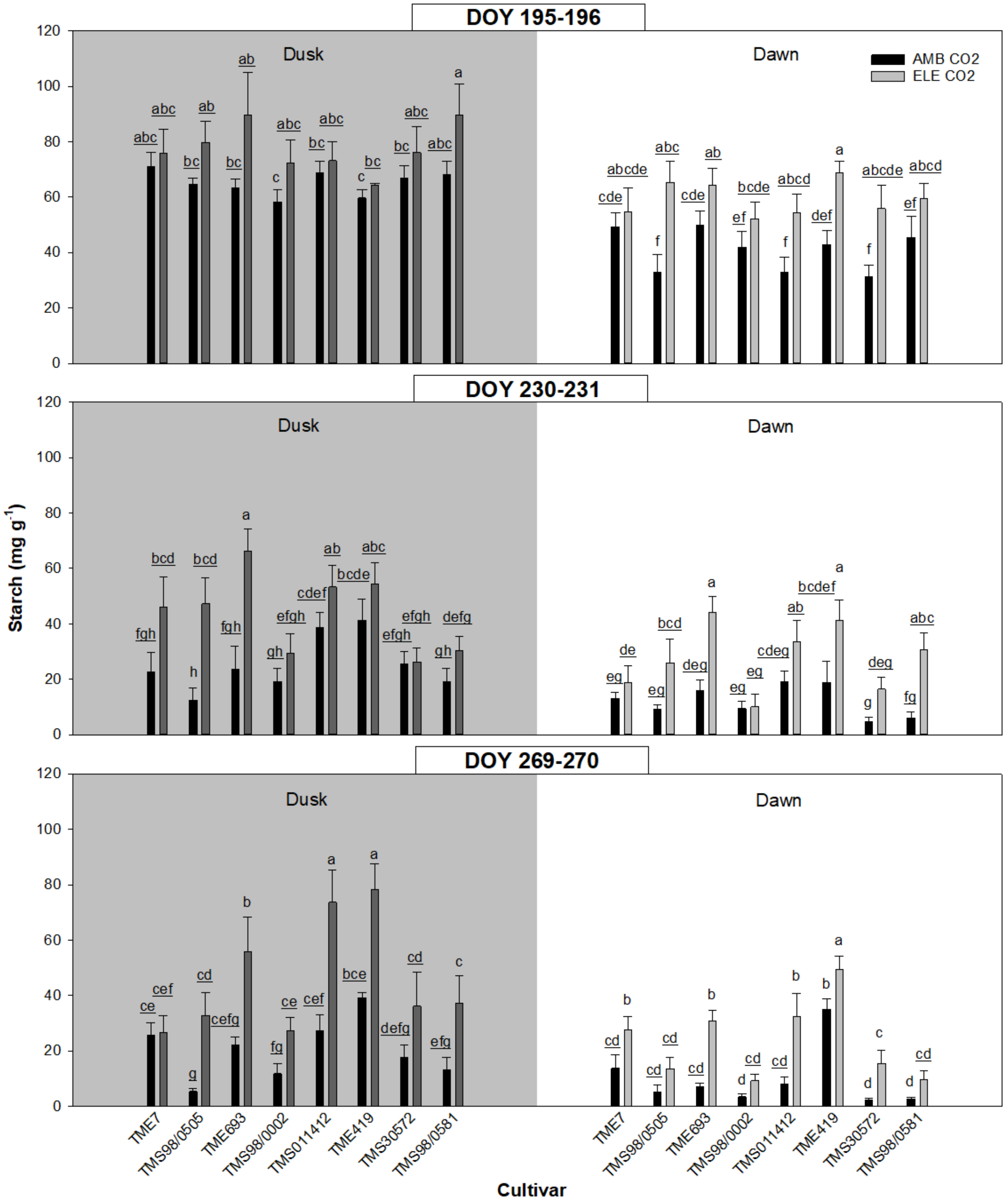

Figure S10. Per day average of the starch (mmol m^{-2}) at dusk and dawn from eight cultivars of cassava grown at ambient (AMB CO2) and elevated [CO_2] (ELE CO2). The days of the year (DOY) when the measurements were taken are indicated at the top each of panel. Error bars are

mean ± standard error (SE; n=4). Treatments with different letters represent significant differences (*p*<0.1), underlying is used to help differentiate group of letters.

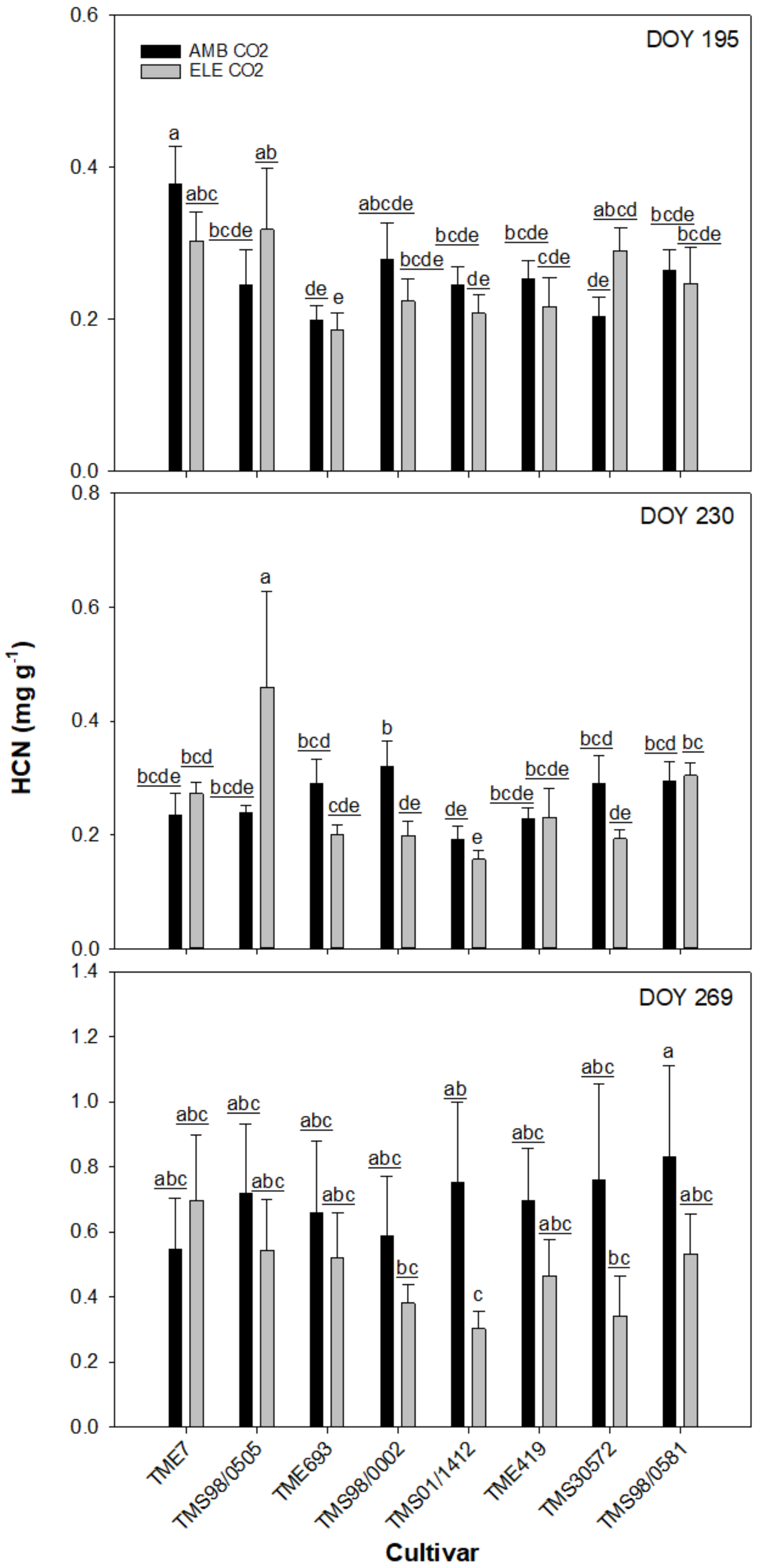

AMB CO2
ELE CO2
DOY 195
DOY 230
DOY 269
HCN (mg g-1)
TME7
TMS98/0505
TME693
TMS98/0002
TMS01/1412
TME419
TMS30572
TMS98/0581
Cultivar

Figure S11. Per day average of the hydrogen cyanide content in leaves (mg g^{-1}) from eight cultivars of cassava grown at ambient (AMB CO2) and elevated [CO_2] (ELE CO2). The days of the year (DOY) when the measurements were taken are indicated in each panel. Error bars are mean ± standard error (SE; n=4). Treatments with different letters represent significant differences (p<0.1), underlying is used to help differentiate group of letters.

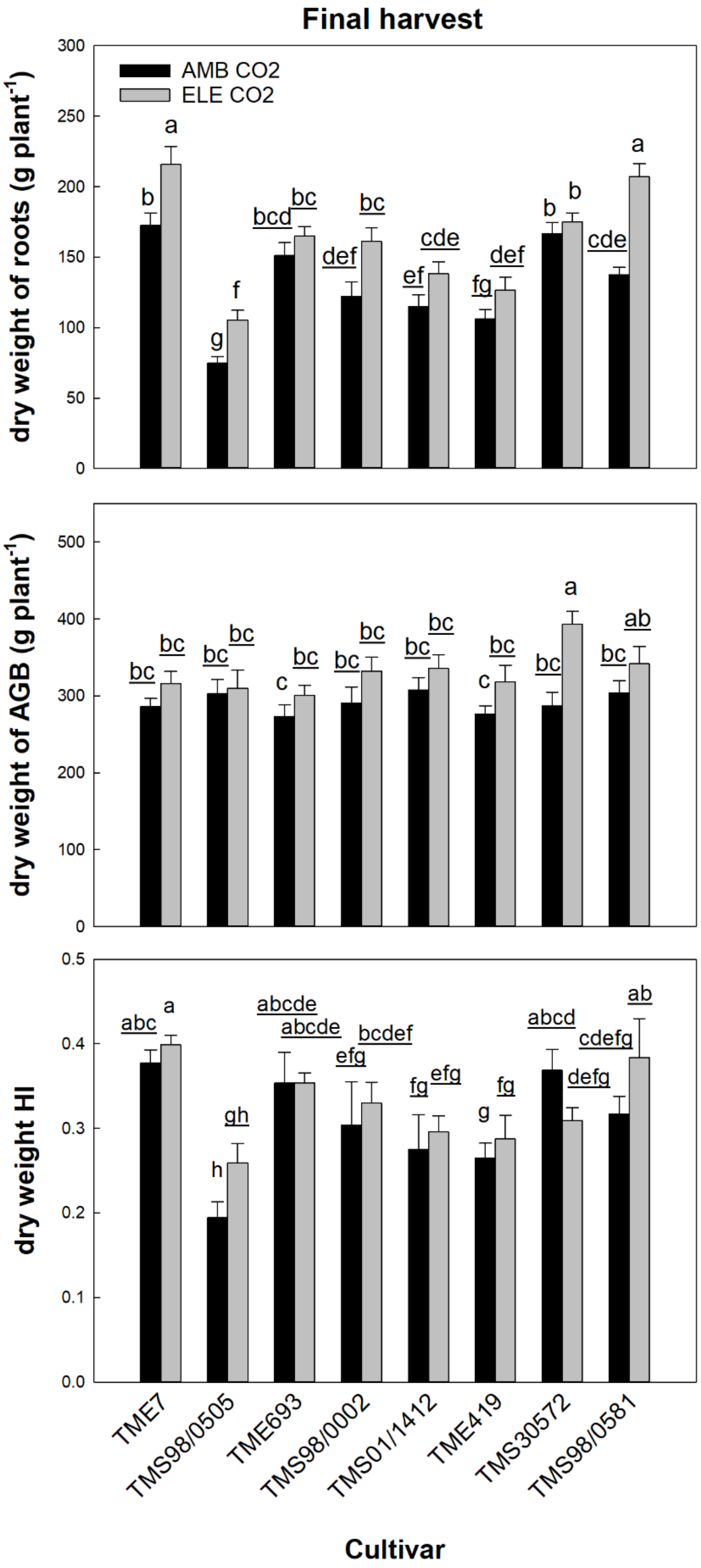
Final harvest
AMB CO2
ELE CO2
dry weight of roots (g plant-1)
dry weight of AGB (g plant-1)
dry weight HI
TME7
TMS98/0505
TME693
TMS98/0002
TMS01/1412
TME419
TMS30572
TMS98/0581
Cultivar

Figure S12. Dry weight from the biomass of eight cultivars of cassava grown at ambient (AMB CO2) and elevated [CO_2] (ELE CO2) during the final harvest. Dry weight of root (g plant^{-1}), dry weight of the above-ground biomass (AGB, g plant^{-1}), and harvest index (HI). Error bars are mean ± standard error (SE; n=4). Treatments with different letters represent significant differences (p<0.1), underlying is used to help differentiate group of letters.

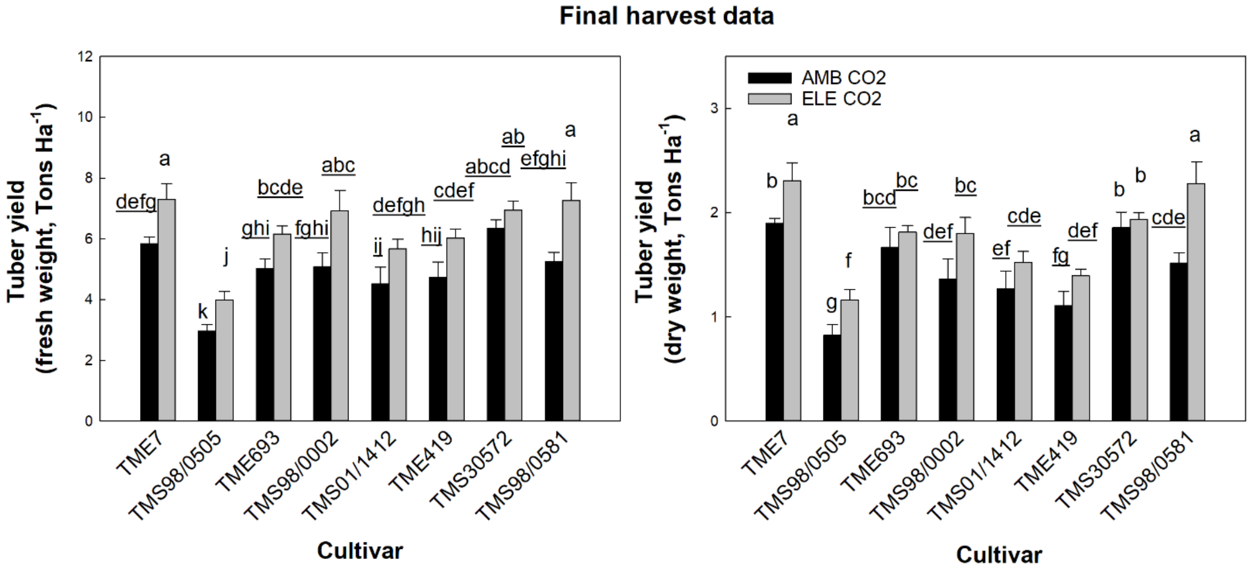

Figure S13. Tuber yield (Tons Ha^{-1}) calculated for the fresh and dry weight of tubers of eight cultivars of cassava grown at ambient (AMB CO2) and elevated [CO_2] (ELE CO2) during the final harvest. Error bars are mean ± standard error (SE; n=4). Treatments with different letters represent significant differences (p<0.1), underlying is used to help differentiate group of letters.

Table S1. Principal component loadings from principal component 1 (PC1) and principal component 2 (PC2) for each one of the parameters considered in this analysis. Values in bold are the main contributors for each PC, and are the only ones represented with an arrow in Figure 2.

**Type of measurements**	**Parameter**	**PC1**	**PC2**
Gas exchange	A	**0.75175**	-0.0163
	g_s	**-0.8527**	-0.0036
	iWUE	**0.92065**	0.08011
	C_i	**0.81698**	-0.0351
	apparent V_{cmax}	-0.7123	0.06686
	apparent J_{max}	-0.5489	0.24197
	g_m	-0.3761	-0.2049
	V_{cmax}	-0.699	0.49735
	J_{max}	-0.3136	0.35618
Plant growth	LAI	0.59478	0.37121
	Height	0.38192	**0.81158**
	Number of leaves in main stem	0.25649	0.65194
	Number of leaves in the whole plant	0.09455	**0.78544**
	Number of branches	0.1098	**0.83738**
	SLA	-0.2481	**0.7911**
Plant material composition	Leaf N	-0.408	-0.6799
	Leaf C:N	0.74265	-0.5719
	Protein	-0.1697	-0.4652
	TSC turnover	-0.327	0.65525
	Starch turnover	0.20133	0.18136
	HCN in leaves	-0.6206	0.21296
	HCN in peel of roots	0.17509	-0.4873
	HCN in core of roots	-0.1774	0.33419
Harvested and yield	Fresh weight roots	**0.76809**	-0.0605
	Fresh weight AGB	0.46195	**0.79477**
	Fresh weight HI	0.47881	-0.4896
	Dry weight roots	0.66134	0.1045
	Dry weight AGB	0.63988	0.35458
	Dry weight HI	0.51376	-0.0008

set of measurements	DOY	Parameters	Main effects		
			$[CO_2]$	cv	$[CO_2]$ x cv
1st	195	*A*	<0.0001	ns	ns
		g_s	0.026	0.041	ns
		iWUE	<0.0001	0.005	0.095
		C_i	<0.0001	0.005	ns
	199-202	apparent V_{cmax}	0.023	ns	ns
		apparent J_{max}	ns	ns	0.038
		g_m	ns	0.024	ns
		V_{cmax}	ns	ns	ns
		J_{max}	ns	ns	ns
2nd	230	*A*	<0.0001	0.005	ns
		g_s	ns	0.077	ns
		iWUE	<.0001	0.014	ns
		C_i	<0.0001	0.012	ns
	226-229	apparent V_{cmax}	ns	ns	ns
		apparent J_{max}	ns	ns	ns
		g_m	ns	ns	0.054
		V_{cmax}	ns	0.040	ns
		J_{max}	ns	ns	ns
3rd	269	*A*	<0.0001	0.049	ns
		g_s	0.033	0.026	ns
		iWUE	<0.001	0.074	ns
		C_i	0.001	0.084	ns
	267-269	apparent V_{cmax}	ns	0.012	ns
		apparent J_{max}	ns	0.036	ns
		g_m	ns	ns	ns
		V_{cmax}	ns	0.030	ns
		J_{max}	ns	ns	ns

Table S2. Statistical analysis (complete block analysis of variance, ANOVA) for the daily average of the following parameters: photosynthetic carbon uptake (*A*, µmol CO_2 $m^{-2}s^{-1}$), stomatal conductance (g_s, mol H_2O $m^{-2}s^{-1}$), intrinsic water use efficiency (iWUE, µmol mol^{-1}), [CO_2] inside the leaf (C_i, µmol mol^{-1}), "apparent" maximum rate of carboxylation by Rubisco (apparent V_{cmax}, µmol m^{-2} s^{-1}) and "apparent" maximum rate of photosynthetic electron transport (apparent J_{max}, µmol m^{-2} s^{-1}), mesophyll conductance (g_m, mol m^2 s^{-1}), V_{cmax} and J_{max}. "Set of measurements" refers to the number of campaigns done to collect the parameters. The main effects are: [CO_2], cultivar (cv), and their interaction. Significant differences ($p<0.1$) and non-statistical significance (ns) are shown in the table.

set of measurements	DOY	Parameters	Main effects		
			$[CO_2]$	cv	$[CO_2]$ x cv
1st	195-196	SLA	0.099	0.002	ns
		leaf N	ns	<0.0001	ns
		C:N	0.044	0.008	ns
		starch dusk	0.007	ns	ns
		starch dawn	<0.0001	ns	ns
		starch turnover	0.006	0.078	ns
		HCN in leaves	ns	0.031	ns
2nd	230-231	SLA	ns	<0.0001	ns
		leaf N	ns	<0.0001	ns
		C:N	<0.0001	<0.0001	ns
		protein dusk	ns	<0.001	ns
		protein dawn	ns	<0.0001	ns
		TSC dusk	0.016	ns	ns
		TSC dawn	ns	<0.0001	0.067
		starch dusk	<0.001	0.002	ns
		starch dawn	<0.0001	0.001	ns
		TSC turnover	ns	0.005	ns
		starch turnover	ns	ns	ns
		HCN in leaves	ns	0.021	0.010
3rd	269-270	protein dusk	ns	<0.001	ns
		protein dawn	ns	ns	ns
		TSC dusk	ns	0.002	0.002
		TSC dawn	0.002	0.063	ns
		starch dusk	<0.0001	<0.0001	0.050
		starch dawn	<0.0001	<0.0001	ns
		TSC turnover	ns	ns	0.039
		starch turnover	0.001	0.061	0.016
		HCN in leaves	0.022	ns	ns

Table S3. Statistical analysis (complete block analysis of variance, ANOVA) for the daily average of the following parameters: leaf area index (LAI, $m^2\ m^{-2}$), height (cm), number of leaves in the main stem and in the whole plant, number of branches, specific leave area (SLA, $m^2\ kg^{-1}$), leaf nitrogen ($g\ m^{-2}$), carbon vs. nitrogen ratio (C:N), protein content (at dusk and dawn; $g\ m^{-2}$), total soluble carbohydrates (TSC, $mmol\ m^{-2}$) and starch ($mg\ g^{-1}$) at dusk and dawn, TSC

and starch turnover, and hydrogen cyanide (HCN) content of leaves, root peel and core (mg g^{-1}). "Set of measurements" refers to the number of campaigns done to collect the parameters. The main effects are: [CO_2], cultivar (cv), and their interaction. Significant differences (p<0.1) and non-statistical significance (ns) are shown in the table.
